# On the parameter combinations that matter and on those that do not: data-driven studies of parameter (non)identifiability

**DOI:** 10.1093/pnasnexus/pgac154

**Published:** 2022-09-14

**Authors:** Nikolaos Evangelou, Noah J Wichrowski, George A Kevrekidis, Felix Dietrich, Mahdi Kooshkbaghi, Sarah McFann, Ioannis G Kevrekidis

**Affiliations:** Department of Chemical and Biomolecular Engineering, Johns Hopkins University, 3400 North Charles Street, Baltimore, MD 21218, USA; Department of Applied Mathematics and Statistics, Johns Hopkins University, 3400 North Charles Street, Baltimore, MD 21218, USA; Department of Mathematics and Statistics, University of Massachusetts, 710 N Pleasant St, Amherst, MA 01003, USA; Department of Informatics, Technical University of Munich, Boltzmannstr. 3, Garching 85748, Germany; The Program in Applied and Computational Mathematic, Princeton University, Washington Road, Princeton, NJ 08544, USA; Department of Chemical and Biological Engineering, Princeton University, 50–70 Olden St, Princeton, NJ 08544, USA; Lewis-Sigler Institute for Integrative Genomics, Princeton University, Princeton, NJ 08540, USA; Department of Chemical and Biomolecular Engineering, Johns Hopkins University, 3400 North Charles Street, Baltimore, MD 21218, USA; Department of Applied Mathematics and Statistics, Johns Hopkins University, 3400 North Charles Street, Baltimore, MD 21218, USA

**Keywords:** parameter nonidentifiability, manifold learning, model order reduction, data mining

## Abstract

We present a data-driven approach to characterizing nonidentifiability of a model’s parameters and illustrate it through dynamic as well as steady kinetic models. By employing Diffusion Maps and their extensions, we discover the minimal combinations of parameters required to characterize the output behavior of a chemical system: a set of *effective parameters* for the model. Furthermore, we introduce and use a Conformal Autoencoder Neural Network technique, as well as a kernel-based Jointly Smooth Function technique, to disentangle the *redundant* parameter combinations that do not affect the output behavior from the ones that do. We discuss the interpretability of our data-driven effective parameters, and demonstrate the utility of the approach both for behavior prediction and parameter estimation. In the latter task, it becomes important to describe level sets in parameter space that are consistent with a particular output behavior. We validate our approach on a model of multisite phosphorylation, where a reduced set of effective parameters (nonlinear combinations of the physical ones) has previously been established analytically.

Significance Statement A mathematical model is *nonidentifiable* if observations of its output behavior do not suffice to uniquely determine the parameter values resulting in these observations. We propose a fully data-driven approach to distinguish those parameter combinations that affect the behavior (*effective* parameter combinations) from those that have no such influence (*redundant* parameter combinations). We also discuss the interpretability of our data-driven effective parameter combinations by mapping them invertibly to candidate sets of physically interpretable ones. Our scheme performs estimation of effective parameter combination values from observations, foliation of parameter space by observation level sets, as well as behavior estimation/prediction from parameters in a seamless, data-driven manner.

## Introduction

Model reduction has long been an important endeavor in mathematical modeling of physical phenomena and, in particular, in the modeling of large, complex kinetic networks of the forms that arise in combustion or in cellular signaling (1–3). A rich array of techniques, often based on time-scale separation, exist that can result in a smaller number of *effective state variables* and, consequently, a reduced set of coupled nonlinear differential equations [e.g. Benner et al. (4), Quarteroni et al. (5), and from our work (6–10)]. Yet, it also becomes important to discover, when possible, a smaller number of *effective parameters*. These are (possibly nonlinear) combinations of the original, usually physically meaningful, model parameters on which the output behavior depends. A universally accepted and practiced approach toward reducing the set of parameters, undertaken before any computation is, of course, dimensional analysis (11).

Beyond dimensional analysis, the issue of parameter nonidentifiability, whether truly structural or approximate, has been the subject of extensive studies for decades, with rekindled interest in recent years (12,13). Such developments are eloquently summarized in ref. (14). This can be attributed in part to sloppiness/MBAM studies (15, 16); the study of active subspaces (17); the increased availability and exploitation of symbolic regression packages (18); and, more generally, to recent advances in data science and manifold learning techniques (19, 20). To a large extent, established model reduction techniques hinge on the availability of analytical model equations and operations (e.g. singular perturbation theory-based expansions) on these closed form equations.

This work aspires to synthesize and implement a purely data-driven process for finding reduced effective parameters. The type of models we consider here are systems of coupled, nonlinear, first-order differential equations describing time-evolution of chemical/biochemical reaction networks, but the approach is applicable more generally to the parameterization of input–output relations. Here, the inputs are the parameters, and the outputs are time series of the system observables, such as species concentrations, temperatures, or functions of such quantities.

**Fig. 1.**
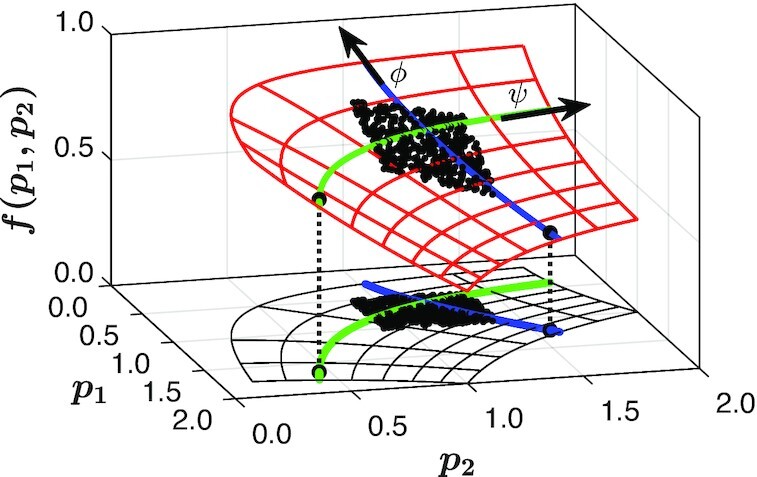
The function *f*(*ϕ*) = exp ( −*ϕ*/2), with *ϕ* = *p*_1_*p*_2_, is sampled at a cloud of points and plotted against the two parameters *p*_1_, *p*_2_ (red mesh). Here, *ϕ* is the *effective parameter*, which we call the “meaningful” parameter combination. The green curve indicates a level set of this effective parameter, for which *f*(*ϕ*) − *C* = 0, for some constant *C* (here, *C* = 0.75). The blue curve illustrates the direction orthogonal to each level set of *ϕ*, parameterized by $\psi =p_1^2-p_2^2$, which we call the “redundant” parameter combination because it does not affect the output. The projection onto the (*p*_1_, *p*_2_)-plane helps illustrate the level sets of the meaningful and redundant parameter combination(s) in parameter space.

In Fig. 1, we illustrate a simple model with structurally nonidentifiable parameters. The model output, *f*(*p*_1_, *p*_2_) = exp (−*p*_1_*p*_2_/2), though plotted as a function of the two parameters (*p*_1_, *p*_2_), in fact depends only on their product *ϕ* = *p*_1_*p*_2_. The output data do not suffice to identify or estimate *p*_1_ and *p*_2_ independently: observations can only confine pairs of *p*_1_ and *p*_2_ to a level set, colored green in Fig. 1, of the *effective parameterϕ*. It is interesting to observe that these level sets are parameterized by the quantity $\psi =p_1^2-p_2^2$, which is conformal everywhere to *p*_1_*p*_2_, thus making *ϕ* and *ψ* an orthogonal system of coordinates (cf. polar or hyperbolic coordinates). A level set for *ψ* is colored blue in Fig. 1. This is the parameter combination that *does not matter to the output*, one that is “redundant”: keeping the output constant while changing *ψ* traces out the level set *ϕ* = *C*. To trace out the possible values of the output, one could of course fix one parameter (say, *p*_2_) and vary the other(s). In that case, however, the sensitivity of the output to the variation of *p*_1_ depends on the value at which we choose to keep *p*_2_ constant. This variability is avoided when using a conformal orthogonal set of coordinates, such as the one in the figure.

In our illustrative models, the system is available in the form of a “black box” set of ordinary differential equations (ODEs): given parameter values and initial conditions, one can record time series of the evolution of the system states, or of functions of the system states. But the evolution equations are not explicitly available, so that analytical (possibly perturbative) approaches to reduction of either system states or parameters (outputs or inputs) cannot be undertaken. Given such an input–output model, we start by systematically prescribing a set of numerical experiments for data collection. These data will be processed with manifold learning techniques—here, Diffusion Maps (DMaps) and Geometric Harmonics (GH)—as well as their extensions: output-informed DMaps and Double DMaps GH. Processing the data will:

- determine the number of model parameter combinations that matter, i.e. the meaningful effective parameters that affect the model output;
- consequently, determine the number of model parameter combinations that do not matter, the redundant ones;
- interpret the meaningful parameter combinations through computational testing/validation of expert suggestions, or possibly through symbolic regression;
- disentangle the redundant parameter combinations from the meaningful effective ones (21, 22), which is accomplished using deep learning techniques (Conformal Autoencoders) or, alternatively, kernel-based Jointly Smooth Feature extraction (23);
- translate between the data-driven effective parameters and physical ones, which underscores the importance of level sets in parameter space consistent with the same output behavior.

We believe these capabilities constitute a useful toolkit for data-driven reparameterization of models, whether computational or physical/experimental. In the experimental setting, the same toolkit can be applied; one will perturb (“jiggle”) all inputs/parameters around a base point, record the richness of the resulting output behavior, and establish (through the same framework) correlations between parameters’ richness and output richness—quantify it and parametrize it.

The remainder of the paper is organized as follows: In the section “The MSP model,” we will demonstrate and visualize the discovery of the intrinsic dimensionality of the meaningful effective parameter space through our main illustrative example: a six-equation multisite phosphorylation (MSP) kinetic model and its analytical reduction by Yeung et al. (24). In the section “Data-driven parameter reduction,” we compare our data-driven effective parameter constructs with those previously obtained analytically and discuss their interpretability, both numerically and through symbolic regression. Finally, we demonstrate the use of these effective parameters in behavior prediction for new physical parameter settings in the section “Effective parameter identification” and (a type of) parameter estimation for previously unobserved behaviors in the section “Behavior estimation.” Toward the latter task, in the section “Parameter estimation,” we discover and parameterize entire level sets in parameter space that are consistent with this new observed behavior; this requires discovering the redundant parameter combinations. In the section “On the parameter combinations that do not matter,” a deep learning architecture (Conformal Autoencoder Networks) as well as an alternative kernel-based *Jointly Smooth Functions (JSFs) extraction* is used for this task of disentangling meaningful effective parameters from redundant ones. We conclude by summarizing the approach and offering a discussion of its potential, shortcomings, and current research directions.

In Supplementary Material Sections S5 and S6, we have included two additional examples. The first comes from a textbook nonidentifiable dynamical system representing a compartmental model and the second is a steady-state example, which allows us to illustrate how our data-driven framework behaves when transitions between qualitatively distinct behavior regimes arise as one traverses the original parameter space.

## Results

The “black box” models that we seek to parameterize in our data-driven work arise mainly from chemical kinetic mechanisms (e.g. Eq. 1), which give rise to systems of ODEs for the evolution in time of the species concentrations as output, depending on several kinetic parameters, possibly including the total quantity of a catalyst or enzyme, as input. In certain parameter regimes, the existence of disparate (fast–slow) time scales allows one to explicitly reduce a detailed kinetic scheme through, e.g. the Bodenstein (25) or Quasi-Steady-State Approximation (QSSA) to an effective reduced one, characterized by new, reduced effective parameters.

The detection of such effective parameters in our scheme will be achieved by using the manifold learning algorithm DMaps (19), for which a more detailed description is given in Section S2A of the Supplementary Material. We will illustrate that, given a systematically collected data set, and with an appropriate *metric*, DMaps can be used for parameter reduction: discovery of effective parameter combinations that affect the output, as well as parameter combinations that do not affect it. The motivation for our work arose from studying the reduction of the following MSP model.

### The MSP model

Yeung et al. (24) proposed and analyzed a kinetic model that describes the dual phosphorylation of extracellular signal-regulated kinase (ERK) by an enzyme known as mitogen-activated protein kinase kinase (MEK). Here, ERK can exist in any of three states: *S*_0_, *S*_1_, and *S*_2_, where the subscript indicates the number of times the substrate has been phosphorylated. The MEK enzyme, denoted by *E*, forms complexes *ES*_0_ and *ES*_1_ with the first two phosphostates. The reaction mechanism for this system is given by

(1) \begin{eqnarray*} && {\mathit{ E}} + {\mathit{ S}_0} \begin{array}{c}k_{f,1}\\\rightleftharpoons \\k_{r,1} \end{array} {\mathit{ ES}_0} \begin{array}{c}{\rm k_{cat,1}}\\\rightarrow \\{} \end{array}\mathrm{\mathit{ ES}_1} \begin{array}{c}{\rm k_{cat,2}}\\\rightarrow \\{} \end{array} \mathrm{\mathit{ E}} + \mathrm{\mathit{ S}_2}\nonumber \\&& \qquad \qquad \qquad \qquad k_{f,2} \upharpoonright \downharpoonright k_{r,2}\nonumber \\&& \qquad \qquad \qquad \qquad \mathrm{E} + \mathrm{S_1}\;, \end{eqnarray*}

with the six rate constants comprising our vector of inputs/parameters:

$$\boldsymbol{p}=[k_{\rm{f},1},\, k_{\rm{r},1},\, k_{\rm{cat},1},\, k_{\rm{f},2},\, k_{\rm{r},2},\, k_{\rm{cat},2}]^\top \in \mathbb {R}^6.$$

The governing system of ordinary differential equations is listed in Section S1A of the Supplementary Material.

Yeung et al. used the QSSA for the species *ES*_0_ and *ES*_1_ along with stoichiometric conservation to approximately reduce the above system: If the assumptions

$$\begin{eqnarray*}
S_{\rm{tot}}\ll \frac{k_{\rm{f},1}+k_{\rm{cat},1}}{k_{\rm{f},1}},\qquad S_{\rm{tot}}\ll \frac{k_{\rm{r},2}+k_{\rm{cat},2}}{k_{\rm{f},2}}
\end{eqnarray*}$$

reasonably hold, where

$$S_{\rm{tot}}=\left.[{\rm S_0}]\right|_{t=0}=[{\rm S_0}]+[{\rm S_1}]+[{\rm S_2}]+[{\rm ES_0}]+[{\rm ES_1}],$$

then the initial model reduces to a three-state linear kinetic model that depends on only three effective parameters, which are combinations of the full model parameters

(2) $$\begin{eqnarray*}
\kappa _1 & =&[{\rm E}]\, \frac{k_{\rm{f},1}k_{\rm{cat},1}}{k_{\rm{r},1}+k_{\rm{cat},1}},\nonumber \\\kappa _2 & =&[{\rm E}]\, \frac{k_{\rm{f},2}k_{\rm{cat},2}}{k_{\rm{r},2}+k_{\rm{cat},2}},\nonumber \\\pi & =&\frac{k_{\rm{cat},2}}{k_{\rm{r},2}+k_{\rm{cat},2}}.
\end{eqnarray*}$$

The reduced equations can be found in Section S1B of the Supplementary Material. We will attempt to derive such a reduced parameterization in a data-driven manner.

### Data-driven parameter reduction

We select a *base point* in parameter space

$$\begin{eqnarray*}
\tilde{\boldsymbol{p}}=[0.71,\, 19,\, 6700,\, 9200,\, 0.97,\, 5200]^\top ,
\end{eqnarray*}$$

which is situated in the region of parameter space where the reduction assumptions hold. We select a reference initial condition [*S*_0_] = 5 and [*E*] = 0.66, with the other species not initially present. Numerically integrating the associated system of ODEs, we collected 10,000 dynamic observations of the system output in response to perturbations of each parameter within $\pm 10\%$ of its base value. Note that our random parameter perturbations, are merely a device for sampling the neighborhood of the base point in input/parameter space; a grid of equally spaced points would also suffice.

In the following analysis, we take as our outputs the concentration [*S*_2_] at *t* ∈ {2, 4, …, 20}, which yields a 10D observation vector at each parameter setting. For this example, the choice of [*S*_2_] as the observed output is not particularly significant; the temporal response of *any* time-varying chemical species or combination thereof would give the same results [based on Takens’ embedding theorem (26)]. We will refer to this particular data set **X** as the *transient data*. This data set samples what in the literature is referred to as *the model manifold*, whose dimensionality determines the number of meaningful (effective) parameters (16, 27, 28).

A second data set, **Y**, was obtained through computational optimization experiments, in which we estimated vectors of six parameter values that best fit the reference transient, we obtained at the base parameter setting. In these experiments, initial conditions were chosen randomly from a log10-uniform distribution, with lower and upper bounds set, respectively, at 10^−3^ and 10^+3^ times the rates estimated by Aoki et al. (29). We performed nonlinear least-square fits of these transients from 1,000 random initial conditions in 6D parameter space, as described in ref. (24). Upon successful completion of these computations, we have 1,000 6D “optimal fits” of the base parameter setting; we call this data set the *optimization data*.

We first computed *output informed* DMaps, with the distance metric described in Section S2A of the Supplementary Material, on the transient data set. The observed outputs in **X** for these computations, are used without reference to the values of the analytical parameters provided in Eq. (2); the latter will only be considered later for validation of our data-driven approach: comparing our effective parameters to previously analytically known ones will confirm that our data-driven scheme finds a parameter representation that is equivalent to that proposed in Eq. (2), which we use later only as a means of comparison to confirm that our data-driven scheme finds a representation that is equivalent to that proposed in ref. (24). The number of independent/nonharmonic eigenvectors indicates the effective dimensionality of the model manifold. We found three nonharmonic DMaps eigenvectors (30)

$$\boldsymbol{\phi }=(\phi _1,\phi _3,\phi _9)\in \Phi \subset \mathbb {R}^3$$

and deduced that the intrinsic dimensionality of the transient data set, and thus of the model manifold, is three. We then turned to the optimization data set and performed both principal component analysis (PCA) and “regular” DMaps. We found that the intrinsic dimensionality of the optimization data set is also three, whether we estimate it from PCA or from DMaps. These two results corroborate/complement each other, since three plus three equals six, the total number of original parameters.

**Fig. 2.**
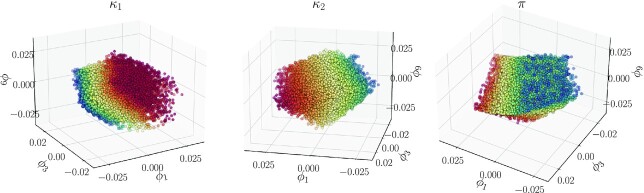
The first three independent, nontrivial eigenvectors, $\boldsymbol{\phi }_1,\boldsymbol{\phi }_3,\boldsymbol{\phi }_9$, colored by (computed) values of the three theoretical effective parameters, *κ*_1_, *κ*_2_, π, respectively, for a transient data set.

The dimensionality of the *transient data set* could be estimated from the dimension of the null space of either the sensitivity matrix or the sensitivity Fisher information matrix (14) at the base point. Beyond this estimate, however, our approach discovers a *global* parameterization over the data of the output in terms of $\boldsymbol{\phi }=(\phi _1,\phi _3,\phi _9)$, which are our data-driven effective parameters. These eigenmodes capture the directions, in full parameter space, that matter to the output: the parameter changes that affect the response of our system. Figure 2 illustrates these three leading nonharmonic eigenvectors, colored by the analytical effective parameters of Yeung et al. in Eq. (2). Even though it is difficult to visually appreciate a 3D point cloud through color, we believe one gets a clear visual impression that the data-driven effective parameter set and the analytical effective parameter set are one-to-one with each other. We will quantify this below.

We remind the reader that the DMaps effective parameters, like the analytical ones, will in general correspond to *combinations* of the original parameters. But while the analytical effective parameters are physically explainable [Eq. (2) shows their dependence on the original parameters], no such a priori physical interpretation comes with the proposed data-driven effective parameters. We will address this issue below.

**Fig. 3.**
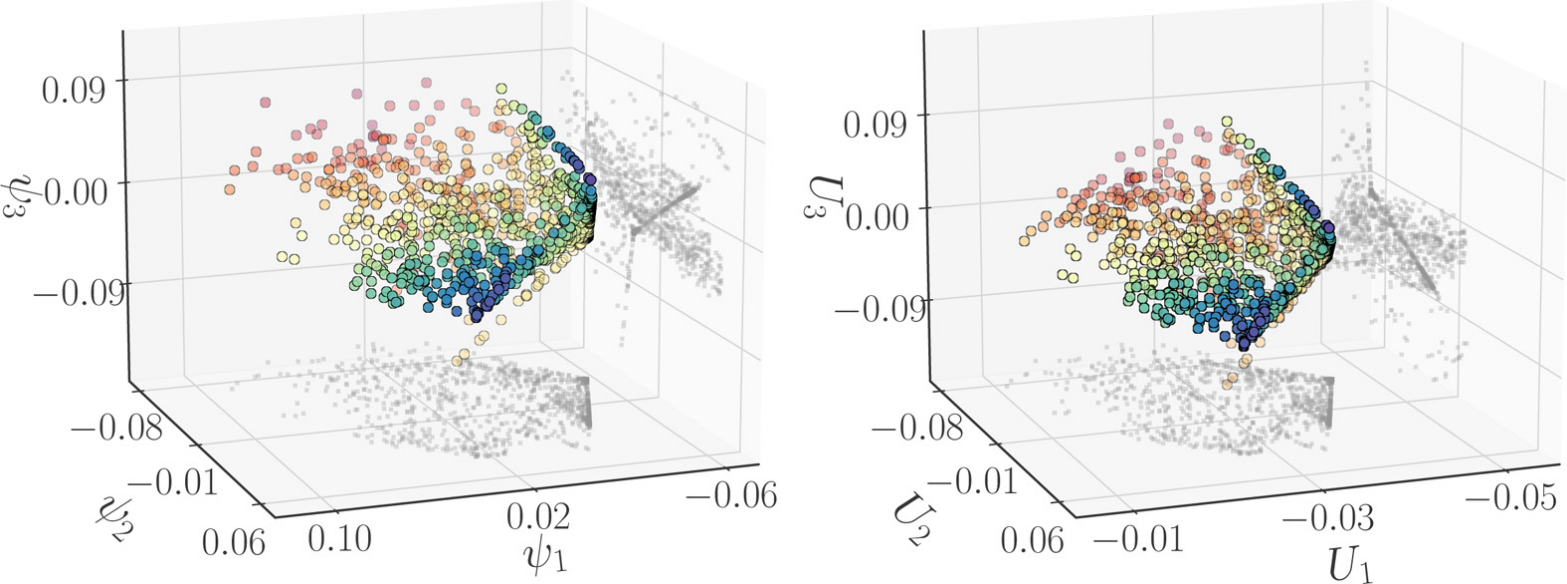
(Left) Independent eigenvector coordinates, *ψ*_1_, *ψ*_2_, *ψ*_3_, for the optimized data set, colored by *ψ*_2_. (Right) The three dominant singular vectors computed with PCA, colored by the second, *U*_2_.

Computing DMaps on the *optimization* data also results in an intrinsically 3D parameterization of the manifold of equivalent optima (Fig. 3). The intrinsic parameters computed for this data set uncover the directions in parameter space that produce (approximately) the same response: the reference trajectory at the base input settings. This dictates how many parameter combinations *do not matter* to the recorded output response. This structural nonidentifiability, computed around a selected output response (one in a base setting) is a property of the system in a neighborhood of that setting, as long as the intrinsic dimensionality of the responses does not change when we perturb the base parameter values (i.e. as long as the QSSA remains valid, see the discussion in Section S2D of the Supplementary Material). For our example, it was sufficient to perform *linear* data processing of the optima by PCA. Indeed, the three redundant parameter combinations for the reference trajectory happen to live on a 3D hyperplane in full parameter space; this hyperplane contains $\sim \!99\%$ of the total variance of the 6D parameter vectors in the optimization set. In this example, it so happens that linear data analysis (PCA) is sufficient to determine the “minimal response richness”: The responses lie on a 3D hyperplane in the 10D output space. In general (and, we expect, most often), PCA will suggest *more* than the truly minimal number of effective parameters to span the data, and nonlinear tools like DMaps would be required to find a minimal set.

We already have our first result: a data-driven corroboration of the *number* of effective parameter combinations. Three of them matter, and three of them do not, adding up to the correct total number of six full inputs. The reader may already have noticed that these structurally unidentifiable combinations *are not global*; they are valid only for the reference trajectory. Beyond finding this number, we will also construct a global parameterization/foliation of the “hypersurfaces that do not matter” in the original input space. Even though only three-dimensional, they are impossible to visualize, leading to our introduction of a visualizable caricature below.

### Effective parameter identification

The leading nonharmonic eigenvectors, $\boldsymbol{\phi }$, computed for the transient data **X** provide an intrinsic parameterization of this data set, i.e. a set of coordinates parameterizing the model manifold (see the discussion on Section S2A of the Supplementary Material for clarification of the term nonharmonics). However, they are not necessarily physically meaningful. In order to interpret them, the data scientist who knows their dimensionality can now ask a domain scientist to suggest a set of physically meaningful parameter combinations, *κ_i_*, and try to quantitatively establish a one-to-one correspondence between the data-driven *ϕ*_*i*_ and the hypothesized meaningful *κ_i_*. This approach to interpretability has been proposed and used in refs. (31–34) for the case of data-driven effective *variables*, and it can be extended, as we propose here, for data-driven effective *parameters*.

In our case, Yeung et al. have already provided us with good candidate analytical effective parameters $\boldsymbol{\kappa }=(\kappa _1,\kappa _2,\pi )\in {K}\subset \mathbb {R}^3$. We seek a (hopefully smooth) invertible mapping *f*: Φ → *K* from the DMaps space to the space of analytical effective parameter values and back. This mapping is constructed through a “slight twist” on GH, which we call Double DMaps, explained in Section S2C of the Supplementary Material. From the total 10,000 collected data points, we use 7,000 as training points and 3,000 as test points for our Double DMaps. We use the inverse function theorem (IFT) described in Section S2E of the Supplementary Material to check that our data-driven effective parameters are indeed *locally* one-to-one with the known analytical effective parameters (Eq. 2). We then use our Double DMaps GH to express the three theoretical effective parameters $\boldsymbol{\kappa }=(\kappa _1,\kappa _2,\pi )\in {K}\subset \mathbb {R}^3$ as (approximate) functions of our coordinates $\boldsymbol{\phi }$.

**Fig. 4.**
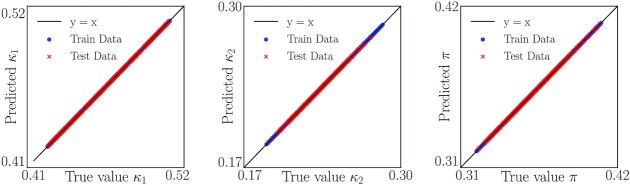
The three theoretical effective parameters predicted as a functions of the DMaps eigenvectors $\boldsymbol{\phi }$ with Double DMaps. (Left) $\kappa _1=f_1(\boldsymbol{\phi })$, (middle) $\kappa _2=f_2(\boldsymbol{\phi })$, and (right) $\pi =f_3(\boldsymbol{\phi })$. Blue dots denote the training points (7,000 data points) and red crosses the test points (3,000 data points).

An alternative realization of this map (data-driven effective to analytical effective) *and its inverse* can also be constructed through the “technology” of neural networks: We used the data-driven effective parameters as inputs in a neural network whose outputs are the analytical effective parameters. Specifically, we used a five-layer, fully connected network with 30 nodes per layer and tanh  activation functions, which we optimized via ADAM to achieve a mean squared error (MSE) on the order of 10^−6^. Training this network provides an alternative realization of the mapping between the data-driven *ϕ*_*i*_ and the interpretable (here analytically obtainable) *κ_i_*, the map *f*: Φ → *K*. We also obtained the inverse map, *f*^−1^: *K* → Φ, by training a second neural network that implemented the same architecture and training scheme but with inputs and outputs switched. Instead of training two separate networks, one could combine the two networks into an autoencoder. Being able to construct the forward and the inverse mapping confirms the global one-to-one correspondence of the two sets on the data: The autoencoder would not be trainable otherwise. Figure 4 plots the ground truth values of the three effective parameters against those interpolated with GH.

**Fig. 5.**
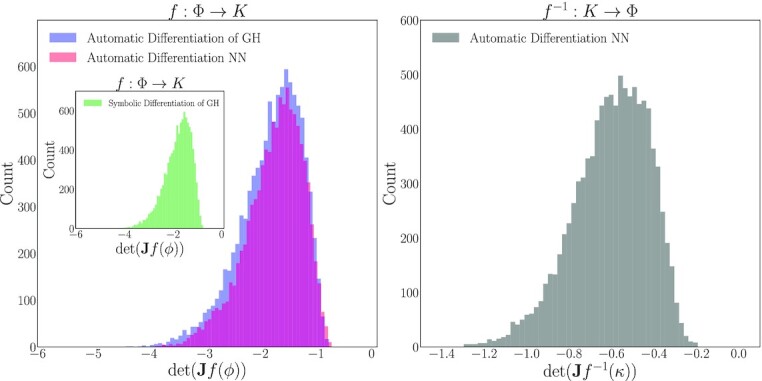
(Left) Histograms of the determinant of the Jacobian, $\det \, \mathbf {J}f(\boldsymbol{\phi })$, computed on each observed data point with automatic and symbolic differentiation of GH and with automatic differentiation using a neural network. (Right) Histogram of Jacobian determinants for the inverse function, $\det \, \mathbf {J}f^{-1}(\boldsymbol{\kappa })$, computed with a neural network.

To establish that this map *f*: Φ → *K* is invertible, we first confirm that the determinant of its 3 × 3 Jacobian matrix is bounded away from zero for all points in our data set. By construction, *f* is continuously differentiable, so the IFT guarantees local invertibility in a neighborhood of any point $\boldsymbol{\phi }\in \Phi$, where the Jacobian matrix

$$\begin{eqnarray*}
\mathbf {J}f(\boldsymbol{\phi })=\left[\begin{array}{ccc}\partial \kappa _1/\partial \phi _1 & \quad \partial \kappa _1/\partial \phi _3 & \quad \partial \kappa _1/\partial \phi _9 \\\partial \kappa _2/\partial \phi _1 & \quad \partial \kappa _2/\partial \phi _3 & \quad \partial \kappa _2/\partial \phi _9 \\\partial \pi /\partial \phi _1 & \quad \partial \pi /\partial \phi _3 & \quad \partial \pi /\partial \phi _9\end{array}\right]
\end{eqnarray*}$$

is nonsingular. In Fig. 5, we illustrate that $\det \mathbf {J}f(\boldsymbol{\phi })$ is bounded away from zero on our complete data set of 10^4^ points. Furthermore, our success in training the decoder component indicates that $f:\Phi \rightarrow \boldsymbol{K}$ is globally invertible over our data and that our computed data-driven effective parameters are indeed one-to-one with the proposed theoretical ones [Eq. (2)].

The effective parameters proposed in ref. (24) were obtained by applying the QSSA to the full model. Simply by rearranging and simplifying the terms in Eq. (2), we could derive another equally plausible triplet of effective parameters:

(3) $$\begin{eqnarray*}
\mu _1^{\prime } =[{\rm E}]\frac{k_{\rm{f},1}k_{\rm{cat},1}}{k_{\rm{r},1}+k_{\rm{cat},1}} \quad \mu _2^{\prime } =\frac{k_4}{k_5k_6},\quad \mu _3^{\prime } =\frac{k_4}{k_6}.
\end{eqnarray*}$$

**Fig. 6.**
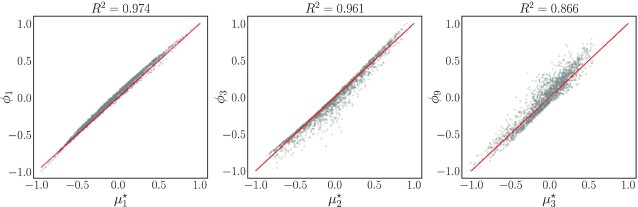
The three eigenvectors, $\boldsymbol{\phi }$, are fitted as functions of the original parameters, $\boldsymbol{p}$, through a symbolic regression algorithm. Entries of $\boldsymbol{\phi }$ and $\boldsymbol{p}$ were rescaled in the range [− 1, 1]. Expressions for the *μ*^⋆^ are provided in Eq. (4).

Which of the two triplets would a symbolic regression package [e.g. gplearn (35)] select? We illustrate an answer graphically in Fig. 6 and analytically in Eq. (4). Note that, when performing this regression, we rescaled both the original parameters and the DMaps coordinates to lie in the range [−1, 1], as suggested in the package documentation (35):

(4) $$\begin{eqnarray*}
\mu _1^\star & =&0.288(k_{\rm{cat},2}-k_{\rm{cat},1}+k_{\rm{r},2}+k_{\rm{f},1}),\nonumber \\\mu _2^\star & =&0.455(k_{\rm{f},1}-k_{\rm{f},2}),\nonumber \\\mu _3^\star & =&0.218(0.36k_{\rm{f},1}^2-1.38k_{\rm{f},1}k_{\rm{r},2}-k_{\rm{f},2}+k_{\rm{cat},2}\nonumber \\&&-\;k_{\rm{f},1}-k_{\rm{r},2}-0.436),
\end{eqnarray*}$$

where $\mu _i^{\star }$ denotes the *i*th estimated symbolic regression expression/parameter. As illustrated in Fig. 6, these simple linear or quadratic expressions of the original parameters $\boldsymbol{p}$ can fit the coordinates quite accurately. In our opinion, while they can be written down in terms of “simple cognitive basis functions,” (i.e. monomials) ultimately these symbolically regressed parameters are almost as mechanistically uninterpretable as our data-driven effective ones.

### Behavior estimation

Our computational formulation also allows us to obtain a mapping from new values of the effective parameters to the corresponding system output behavior. Each analytical effective parameter *κ*_*i*_ and each element of every observed behavior vector are functions over the intrinsic model manifold, which is parameterized by the data-driven effective parameters *ϕ*_*i*_. If we are given a new triplet of *ϕ_i_*, GH on our Double DMaps can recover any element of any observation vector. If, on the other hand, we are given a new triplet of *κ_i_*, we need only locally invert the known *κ*_*i*_(*ϕ_j_*) functions to the data-driven effective parameters, and proceed as above to predict the corresponding dynamic behavior through GH. Alternatively, after a round of DMaps on the *κ*_*i*_, we perform GH on these DMaps to interpolate any desirable element of the expected behavior vector as a function of the *κ_i_*.

**Fig. 7.**
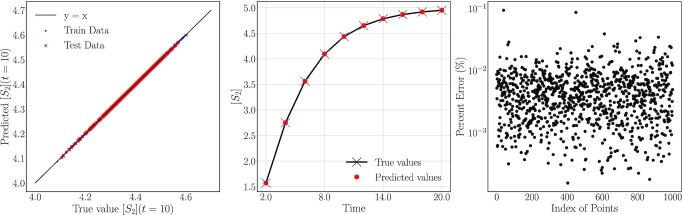
(Left) Comparison of true and predicted values of the product concentration [*S*_2_] at *t* = 10 with our scheme for 4,000 training and 1,000 test points. (Center) A reconstructed concentration profile of *S*_2_ for a test point. With crosses are illustrated the true values, and with red points the values predicted with Double DMaps. (Right) The relative error for the 1,000 unseen behaviors.

To implement this latter procedure, we generated 5,000 triplets of analytical effective parameters by perturbing uniformly within $\pm 20\%$ of the nominal parameter values (*κ*_1_, *κ*_2_, π) = (0.467, 0.232, 0.362), designating 4,000 as training and 1,000 as test points. We used this data set to learn the output concentration profiles for 10 time steps of *S*_2_ with our Double DMaps GH scheme. Figure 7 shows the true values of the concentrations against the predicted values with our scheme for *t* = 10. Across all 1,000 test points for analytical effective parameter values, the relative prediction error does not exceed $0.1\%$.

### Parameter estimation

**Fig. 8.**
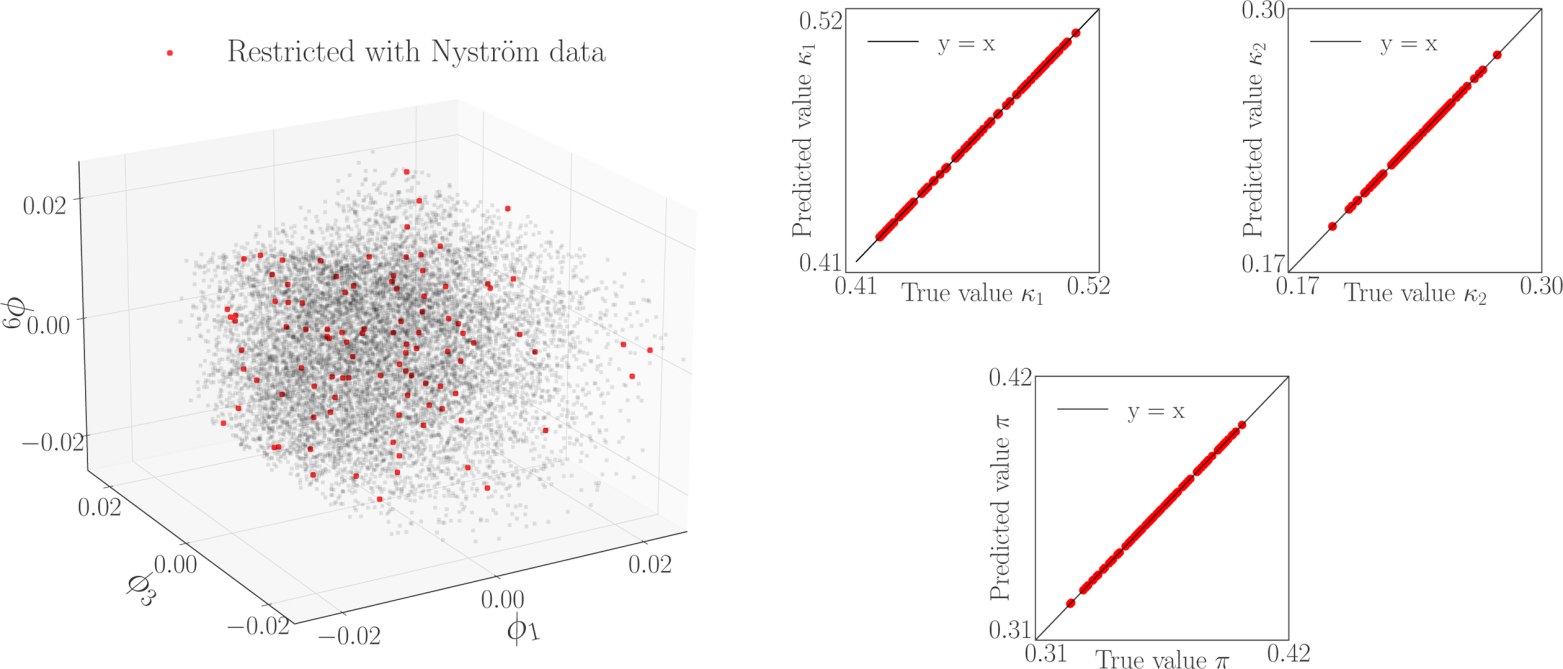
(Left) The unseen behaviors $\boldsymbol{f}(\boldsymbol{p}_{new})$ projected onto DMaps space via the Nyström extension. (Right) For 100 unseen behaviors, the effective parameters (*κ*_1_, *κ*_2_, π) are predicted with our Double DMaps scheme from previously unseen behaviors $\boldsymbol{f}(\boldsymbol{p}_{new})$.

Even when the kinetic mechanism is known, parameter estimation is often challenging, due to measurement noise and differences in the timescales of individual reactions (24). Estimating the parameters not through optimization but through our data-driven scheme is straightforward from a technical standpoint. For previously unseen behaviors $\boldsymbol{f}(\boldsymbol{p}_{\rm new}) = [\rm {\mathit{ S}}_2(\mathit{ t}_{1}|\boldsymbol{p}_{\rm new}),\ldots ,\rm {\mathit{ S}}_2(\mathit{ t}_{f}|\boldsymbol{p}_{new})]$, the Nyström extension (described in Section S2B of the Supplementary Material) directly estimates the corresponding *ϕ_i_* on the model manifold, from which we directly go to the effective parameters $\boldsymbol{\kappa }$ leading to this behavior through our Double DMaps version of GH (see Section S2C). Our approach performs this estimation in the minimal required dimensions—the intrinsic, data-driven ones—that jointly parameterize the observed behavior *and* the meaningful input combinations that produce it. Figure 8 illustrates the projection of 100 previously unseen behaviors to the 3D manifold through the Nyström extension and quantifies how well we can estimate the effective parameters for those unseen behaviors through our scheme.

### On the parameter combinations that do not matter

Having identified a data-driven effective parameterization of the model and constructed data-driven maps from behavior to effective parameters and back, we now need to complete the task by mapping behavior to the original, full parameter set. Clearly, this mapping is not one-to-one: For every observed behavior from the model, there exists *an entire level set* of the original parameter space consistent with this behavior—and with a single set of meaningful parameter combination values. For the optimized data in Fig. 3, we showed that an entire 3D level set exists in the original parameter space, for a given output behavior (and so, for a given set of effective parameters/meaningful parameter combinations). But this does not identify the parameter combinations *that do not matter*, that is, those which do not influence the resulting model behavior as one changes their values by moving along a level set of the effective parameters. In order to describe these level sets, we must employ a data-driven approach that allows us to detect the combinations of original parameters that do not affect the model output. This will disentangle the meaningful effective parameter combinations from the redundant ones. In Fig. 1, this disentangled parameterization was given by *ϕ* ≡ *p*_1_*p*_2_ and $\psi \equiv p_1^2-p_2^2$.

Notice that the level sets of these two types of original parameter combinations are conformal everywhere. Moving *p*_1_ and *p*_2_ along the green level set does not change the model output, whereas moving them on the blue level set suffices to sample all possible output behaviors. In this way, the redundant parameter combinations allow us to construct the set of original, physical parameter values that are consistent with an observed behavior. Alternatively, holding them constant reduces the number of dimensions to be explored when optimizing the model behavior. Finally, after finding a behavior that optimizes a primary objective, the redundant parameter combinations help parameterize the search for an optimal *secondary* objective—not a Pareto multiobjective but rather a *lexicographic* optimization (36). This disentanglement helps outline the nature of this subsequent lexicographic optimization and the dimensions available for it in parameter space. However, since the data are collected locally around the base point, our computation provides only a *springboard* for further systematic exploration. A discussion of the systematic collection of additional data, parsimoniously extending the known “patches” of the level sets is discussed in ref. (37).

#### A visualizable caricature

The 3D level sets of our working MSP example do not lend themselves to visualization. We therefore turn to a simpler kinetic model to illustrate these ideas and methods:

(5) $$\begin{eqnarray*}
\mathrm{S_0 + E} \begin{array}{c}k_f\\\rightleftarrows \\k_r \end{array} \mathrm{ES_0}\begin{array}{c}{\rm k_cat}\\\rightarrow \\{} \end{array} \mathrm{S_1 + E},
\end{eqnarray*}$$

where *S*_0_ and *S*_1_ are two different states of the substrate *S*; *E* is the enzyme; and *ES*_0_ and *ES*_1_ are complexes between the enzyme and the substrate. The differential equations can be found in Section S1C of the Supplementary Material. We chose two base values of the original parameters *k*_f_, *k*_r_, *k*_cat_ to work with. The first base value

$$\boldsymbol{k}_1=(k_{\rm{f}},k_{\rm{r}},k_{\rm{cat}})=(0.71,19,6700),$$

gives a single effective parameter *k*_eff_ ≃ *k*_f_; in Section S3 of the Supplementary Material, we describe the discovery, through our manifold learning, of this single effective parameter and also the construction of its level sets. We choose to discuss here our results for the more interesting case of nominal parameters

$$\boldsymbol{k_2}=(k_{\rm{f}},k_{\rm{r}},k_{\rm{cat}})=(0.97,7000,10000).$$

In this regime, QSSA yields the single effective parameter

(6) $$\begin{eqnarray*}
k_{\rm{eff}}=E_{\rm{tot}}\, \frac{k_{\rm{f}}k_{\rm{cat}}}{k_{\rm{r}}+k_{\rm{cat}}},
\end{eqnarray*}$$

where *E*_tot_ is the total concentration of the enzyme.

**Fig. 9.**
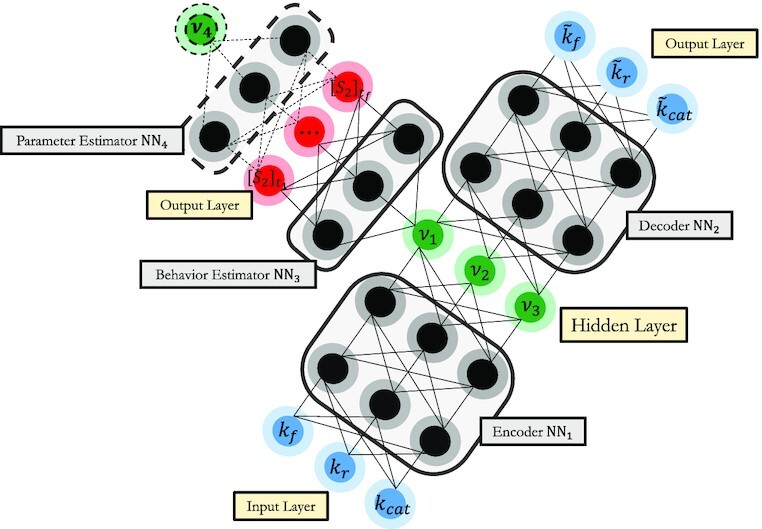
The proposed Y-shaped Conformal Autoencoder consists of the following subnetworks: an Encoder (NN_1_), a Decoder (NN_2_), a Behavior Estimator (NN_3_), and possibly an additional Parameter Estimator (NN_4_) [see Eq. (7)].

We generated 2,000 parameter vectors by sampling each entry uniformly within $\pm 20\%$ of its nominal value. We collected output system behaviors for each parameter vector by integrating the model mechanism of Eq. (5) from the reference initial condition ([*S*_0_], [*E*], [*S*_1_], [*ES*_1_]) = (5.0, 0.66, 0, 0). The response is recorded every 2 seconds in time for five total points per trajectory. Our data-driven approach again detects that the output behavior of the system is intrinsically 1D, and the new single effective parameter *ψ*_1_ is one-to-one with our data-driven effective parameter *k*_eff_, which is a combination of all three original parameters. The level sets of *ψ*_1_ (or *k*_eff_) are 2D curved surfaces (manifolds) in the original parameter space. In order to describe this level set, that is, discover the redundant parameter combinations, we introduced a *Conformal Autoencoder* Y-shaped Neural Network architecture (see Fig. 9).

Our Y-shaped Neural Network scheme consists of several connected subnetworks:

(7) $$\begin{eqnarray*}
&\rm{NN}_1 & :(k_{\rm{f}},k_{\rm{r}},k_{\rm{cat}})\mapsto (\nu _1,\nu _2,\nu _3),\nonumber \\&\rm{NN}_2 & :(\nu _1,\nu _2,\nu _3)\mapsto (\tilde{k}_{\rm{f}},\tilde{k}_{\rm{r}},\tilde{k}_{\rm{cat}}),\nonumber \\&\rm{NN}_3 & :\nu _1\mapsto ([{\rm S_2}]|_{t_1},\ldots ,[{\rm S_2}]|_{t_f}),\nonumber \\&\rm{NN}_4 & :([{\rm S_2}]|_{t_1},\ldots ,[{\rm S_2}]|_{t_f})\mapsto \nu _4.
\end{eqnarray*}$$

We used three multilayer perceptrons illustrated in Fig. 9:

1. “Encoder” (NN_1_), which transforms the original parameters to a reparameterization, disentangling their meaningful combinations (one in the figure) and the redundant ones (two in the figure);
2. “Decoder” (NN_2_), which reconstructs the original parameters; and
3. “Behavior Estimator” (NN_3_), which maps the meaningful combination(s) to the observed output data.

An additional “Parameter Estimator” (NN_4_) could be used to map observed behaviors back to the effective parameter(s) to ensure global invertibility.

The key feature is the loss function, consisting of several parts. The obvious one is the successful reconstruction of the input original parameters (the “Autoencoder” part). Next comes the ability of NN_3_, whose input is the single effective parameter combination we seek, to reproduce the observed output; this forces *ν*_1_ to be one-to-one with the analytically known parameter *k*_eff_. How many output measurements are necessary? Whitney’s (and Takens’) embedding theorems provide guarantees for 2*n* + 1 generic observations, when *n* is the dimension of the model manifold (26). Clearly, to build the architecture, we need to know in advance the number (here, one) of the required meaningful parameter combinations from the dimensionality of the model manifold. This number is the first quantity we compute with our output-informed DMaps analysis of the transient system observations. The third necessary loss component comes from further imposing an *orthogonality constraint* on the Conformal Autoencoder’s latent coordinates $\boldsymbol{\nu }$:

$$\begin{eqnarray*}
\left\langle {\boldsymbol{d\nu }_i,\boldsymbol{d\nu }_j} \right\rangle =0\qquad \forall i\ne j,
\end{eqnarray*}$$

where $\boldsymbol{d\nu }_i$ indicates the vector of partial derivatives of the latent coordinate *ν*_*i*_ in terms of the input parameters (*k*_f_, *k*_r_, *k*_cat_) and 〈 ·, ·〉 indicates the inner product. This constraint is imposed using the automatic differentiation capabilities of the relevant code libraries and aims to disentangle what matters from what does not, making the architecture a “Conformal Autoencoder.” We explain the procedure used to train this Neural Network in Section S2G of the Supplementary Material.

**Fig. 10.**
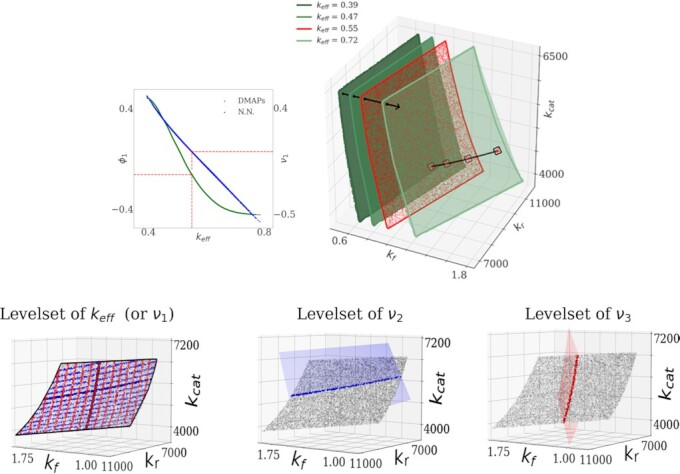
(Top left) The effective parameter *k*_eff_ is one-to-one with the data-driven coordinate *ϕ*_1_, and also with the Neural Network effective variable *ν*_1_. (Top right) The level sets of constant behaviors, the level sets here are surfaces of the form *f*(*k*_f_, *k*_r_, *k*_cat_) = *C*. A particular effective parameter (red point) corresponds to a level set (red surface) of the original parameters (*k*_f_, *k*_r_, *k*_cat_). (Bottom left) The same level set of *k*_eff_ (equivalently, of *ν*_1_, since they are one-to-one), on which the conformal directions are colored as a grid of red and blue lines. (Bottom center) The intersection of the level set of *k*_eff_ with a level set of *ν*_2_. (Bottom right) The intersection of the level set of *k*_eff_ with a level set of *ν*_3_.

We thus discover a parameterization of the two redundant parameter combinations through *ν*_2_ and *ν*_3_. We also discover the Neural Network encoding of the effective parameter, *ν*_1_, which is one-to-one with both *k*_eff_ and *ϕ*_1_ (see Fig. 10). Our Double DMaps can easily approximate the estimation of *ν*_1_ from new, unobserved behavior. Figure 10 shows representative (orthogonally) intersecting level sets of the three *ν_i_*, and the conformal grid of *ν*_2_, *ν*_3_ on a level set of the effective parameter *ν*_1_.

This network can be used to encode a full set of initial parameter values to the effective parameter values that matter and through them to the predicted behavior. More importantly, the already established path from the new, unobserved behavior to the corresponding value of *ν*_1_, the effective parameter that matters, allows us to fix this value as an input to the Decoder NN_2_ and reproduce the level set of original parameters consistent with this new observed behavior by varying the values of *ν*_2_, *ν*_3_.

### JSF extraction

We conclude this section by discussing how a kernel-based method called JSFs, introduced by Dietrich et al. (23), can be extended and used to disentangle input–output relations. Instead of a Neural Network architecture, the “Jointly Smooth Functions” (23) approach, as its name suggests, could be used to find functions of the original parameters and functions of the output measurements that are *jointly smooth* over the available data. Those JSFs between the original parameters and the output are the effective parameters of the model in our case.

**Fig. 11.**
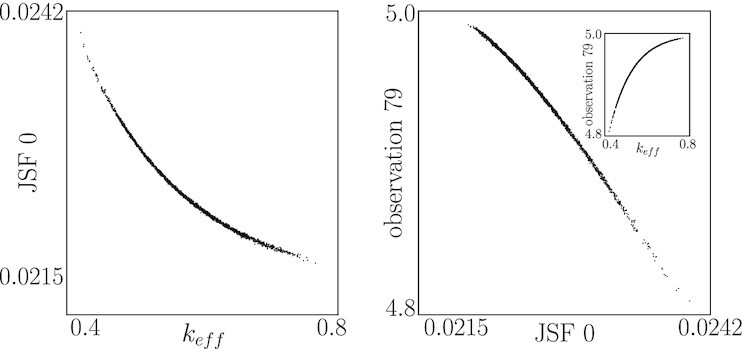
(Left) The first JSF for the second example, compared to the effective parameter *k*_eff_. (Right) The first JSF is one-to-one with the observation $\boldsymbol{x}_1^{(79)}$.

Figure 11 illustrates the results for our second, visualizable example. Two data sets are collected, containing 2,000 samples each. One consists of 20 time-delayed measurements of four output variable observations, (*S*_0_, *S*_1_, *ES*_0_, *E*), which we express as $\boldsymbol{x}_1\in \mathbb {R}^{80}$. The second contains the corresponding parameters $\boldsymbol{x}_2\in \mathbb {R}^3$. We use these two data sets as input to the JSF extraction pipeline (Algorithm in the Supplementary Material) and compute 25 such functions. The first JSF is one-to-one with the known effective parameter *k*_eff_ (bottom left). We additionally plot an output (here, one of the measurements, the 79th one in time) that is also one-to-one with the first JSF (on the right). Note that, to test the robustness of the approach, the latter half of the output measurements were substituted with random noise uniformly distributed over the measurement range.

In our work, we introduce an additional feature of the JSFs that allows to the computation of *redundant* parameter combinations through the JSF approach; this is illustrated in Section S2H of the Supplementary Material.

## Discussion

We have presented a systematic, data-driven approach for obtaining a meaningful reparameterization of parameter-dependent dynamical systems, disentangling the parameter combinations that matter to the output observations (temporal state measurements) from those that do not. The approach is generally applicable to the reparameterization of input–output relations.

We used manifold learning techniques, including DMaps, to jointly parameterize the behaviors observed (the “model manifold”) and the parameter combinations leading to them. We found the minimal number of meaningful parameter combinations (the effective parameters), expressed the outputs as functions of these effective parameters, and showed how to construct data-driven mappings from new effective parameters to the estimated outputs (prediction) and from new output observations back to effective parameters (estimation). It is worth mentioning that, in the case of noisy outputs, the DMaps parametrization will be robust to output noise as long as the scale parameter $\sqrt{\varepsilon }$ remains larger than the amplitude of the noise (19).

Disentangling the parameter combinations that affect the output from those combinations that do not (the redundant parameter combinations) was obtained through a conformal autoencoder neural network. This allows us to now provide, for any observed behavior, not only the effective parameter values for it but also *the level set, in full input parameter space* consistent with this behavior. The capability of disentangling meaningful from redundant by enforcing conformality seems a promising research tool in tasks ranging from data-driven dimensional analysis to the exploration and construction of closures, and to the training of overparameterized neural networks.

We briefly discuss the computational scalability of our approach. Generally, the ambient space dimension of the data influences the computational complexity less than the intrinsic dimension of the model manifold, i.e. the number of effective parameters. The detection of effective parameters in an intrinsically high-dimensional (say, five- or more dimensional) model manifold is less constrained by the scaling of our approach, but hinges on the large amount of data needed to sample the manifold well. Ambient space dimension, i.e. the number of given parameters (including redundant ones) as well as the number of observations, does not matter as much for the computational complexity of our approaches, since DMaps, GH, and JSFs are all based on pair-wise distance matrices that effectively ignore ambient dimension. The computational efficiency of the JSF approach is discussed in ref. (23). In general, kernel-based methods such as DMaps require more careful numerical implementations than Neural Network approaches, otherwise the number of data points becomes a bottleneck. Efficient algorithms that scale to millions of data points, even in high-dimensions, are available; see refs. (23) and (38) for a discussion. Regarding memory, the Conformal Autoencoder network is less demanding than kernel-based approaches, because we can utilize minibatching for training and highly parallelized software with efficient implementations is readily available. The analysis of the computational complexity of the network approach is much more involved than for kernel-based approaches, however, and out of the scope of this paper. Even convergence of the training is not clear, although some recent work hints on global convergence at least in controlled settings (39,40).

It is interesting to consider the interplay of this approach with multiobjective optimization: If some input parameter combinations matter to a dominant objective, while others do not, we can, after a first round of optimization, exploit the redundant parameter combinations and optimize a second, “subservient” objective on optimal level sets of the first, dominant one. This is termed *lexicographic* optimization and can also be related to “lifelong learning.” A conceptually simple example is the training of an overparameterized neural network to perform some task: The primary objective will be the accuracy of the prediction, while the “subservient,” secondary objective can be the pruning of the network for sparsity *while remaining on the level set of successfully optimized predictions*.

Finally, we explored interpretability of our data-driven effective parameters through establishing bijections between them and candidate “tuples” of physical ones, which must come from domain experts. We also explored another simple approach to effective parameter interpretability by symbolically regressing the data-driven effective parameters as functions of the input ones.

This work, creating mappings between parameters (in a sense, inputs to a dynamical system) and observed behavior (outputs), can be extended to create mappings between inputs and states, as well as mappings between states and outputs. We are exploring this direction toward data-driven balanced realizations. We expect that our level set parameterizations of the parameter sets that matter/do not matter (whether through Conformal NN or through JSF computations) may lead to useful extensions of the controllability and observability subspaces of linear theory. In this more general problem formulation, one can go beyond structurally unidentifiable inputs, and uncover spurious observations that are not system outputs (e.g. intrinsic sensor noise in our output observations) (41). We are also exploring JSFs as a promising alternative kernel-based approach. Extracting the components of the inputs and outputs in the jointly smooth directions “that matter” can also help highlight those that do not. A key benefit is that, in addition to removing irrelevant input directions, this computation also removes output directions that are not influenced by the input (parameter) data, and provides a numerically stable and accurate approximation of the function space over the space of the effective parameters.

## Conclusion

We conclude by reiterating that, while the paper was focused on parameter nonidentifiabiity, in a context where the original model parameters function as “inputs” to the model, and the observed state time series are the “output,” our approach is generally applicable to data-driven (re)parametrization of more general input–output relations, with an eye toward disentangling meaningful inputs from redundant ones. Applicability of our current framework in an experimental setting involves (after selection of a reference set of conditions) the systematic local perturbation of all distinct experimental parameters/inputs; data mining on the response/output then leads to the discovery of the meaningful and redundant parameter combinations.

## Supplementary Material

pgac154_Supplemental_Files

## Data Availability

The data underlying this article and the codes used to perform the computations are available in a public repository from the authors at https://gitlab.com/nicolasevangelou/on_the_parameters.

## Previous Presentation

Parts of these results were previously presented at 2021 Virtual AIChE Annual Meeting, November 17, 2021.

## Preprints

A preprint of this article can be found at arXiv:2110.06717.
