# Supplementary material for: On the parameter combinations that matter and on those that do not: data-driven studies of parameter (non)identifiability
Source: PNAS Nexus. 2022 Sep 14;1(4):pgac154. doi: 10.1093/pnasnexus/pgac154 (PMC9802152; doi:10.1093/pnasnexus/pgac154)
Supplement: pgac154_Supplemental_Files [file pgac154_supplemental_files.zip › On_the_Parameters_Supplemental_Info.pdf]

Supplementary Information for

On the Parameter Combinations That Matter and on Those That do Not: Data-Driven Studies of Parameter (Non)identifiability

N. Evangelou, N. J. Wichrowski, G. A. Kevrekidis, F. Dietrich, M. Kooshkbaghi, S. McFann, and I. G. Kevrekidis

Ioannis G. Kevrekidis
yannisk@jhu.edu

This PDF file includes:

Supplementary text
Figs. S1 to S12
Tables S1 to S4
SI References

Supporting Information Text

1. Kinetic Models

A. The MSP Model of Yeung *et al.* We consider the dual phosphorylation of a substrate S by an enzyme E, which is the illustrated mechanism in Equation (1) of the main paper. The substrate can exist in any of three different states (phospostates): S_0, S_1 and S_2, where the index denotes how many times the substrate has been phosphorylated. Using elementary reaction kinetics (1), we derive the following system of first-order differential equations to describe the evolution of the system in time:

$$\frac{d[S_0]}{dt} = -k_{f,1}[E][S_0] + k_{r,1}[ES_0]\,, \qquad [1]$$

$$\frac{d[ES_0]}{dt} = k_{f,1}[E][S_0] - (k_{f,1} + k_{cat,1})[ES_0]\,, \qquad [2]$$

$$\frac{d[ES_1]}{dt} = k_{cat,1}[ES_0] - (k_{r,2} + k_{cat,2})[ES_1] + k_{f,2}[E][S_1]\,, \qquad [3]$$

$$\frac{d[S_1]}{dt} = -k_{f,2}[E][S_1] + k_{r,2}[ES_1]\,, \qquad [4]$$

$$\frac{d[S_2]}{dt} = k_{cat,2}[ES_1]\,, \qquad [5]$$

$$\frac{d[E]}{dt} = -k_{f,1}[E][S_0] + k_{r,1}[ES_0] - k_{f,2}[E][S_1] + k_{r,2}[ES_1] + k_{cat,2}[ES_1]\,, \qquad [6]$$

The conservation laws for substrate and enzyme are given, respectively, by

$$S_{tot} = [S_0]|_{t=0} = [S_0] + [S_1] + [S_2] + [ES_0] + [ES_1]\,, \qquad [7]$$

$$E_{tot} = [E]|_{t=0} = [E] + [ES_0] + [ES_1]\,. \qquad [8]$$

It is worth mentioning, that S_0 and S_1 bind reversibly to the enzyme, which leads to complexes ES_0 and ES_1, respectively. We assume that the experiment begins with all substrate in the S_0 state and all enzyme molecules free. That is, at $t = 0$, we have

$$\begin{bmatrix} [S_0] \\ [ES_0] \\ [ES_1] \\ [S_1] \\ [S_2] \\ [E] \end{bmatrix} = \begin{bmatrix} S_{tot} \\ 0 \\ 0 \\ 0 \\ 0 \\ E_{tot} \end{bmatrix}. \qquad [9]$$

Following the exposition of (2), all concentrations are expressed in micromoles per liter, and the net production rate of each species has units of micromoles per liter per minute.

B. The Reduced MSP Model. If the values of the rate constants place us in the regime where

$$S_{tot} \ll \frac{k_{r,1} + k_{cat,1}}{k_{f,1}}\,, \qquad [10]$$

then we can use the QSSA to obtain the following system of three linear differential equations:

$$\frac{d[S_0]}{dt} = -\kappa_1[S_0] \qquad [11]$$

$$\frac{d[S_1]}{dt} = \kappa_1(1-\pi)[S_0] - \kappa_2[S_1] \qquad [12]$$

$$\frac{d[S_2]}{dt} = \kappa_1\pi[S_0] + \kappa_2[S_1] \qquad [13]$$

where

$$\kappa_1 = [E]\,\frac{k_{f,1}k_{cat,1}}{k_{r,1} + k_{cat,1}}\,, \qquad \kappa_2 = [E]\,\frac{k_{f,2}k_{cat,2}}{k_{r,2} + k_{cat,2}}\,, \qquad \pi = \frac{k_{cat,2}}{k_{r,2} + k_{cat,2}}\,, \qquad [14]$$

are the (analytical) effective parameters proposed in (2), and the initial conditions at $t = 0$ are $[S_0] = S_{tot}$ and $[S_1] = [S_2] = 0$.

C. A Toy Example. The mechanism in Equation (5) of the main text is governed by the system of differential equations

$$\frac{d[\mathrm{S_0}]}{dt} = -k_\mathrm{f}[\mathrm{E}][\mathrm{S_0}] + k_\mathrm{r}[\mathrm{ES_1}] \tag{15}$$

$$\frac{d[\mathrm{ES_0}]}{dt} = k_\mathrm{f}[\mathrm{E}][\mathrm{S_0}] - k_\mathrm{r}[\mathrm{ES_0}] - k_\mathrm{cat}[\mathrm{ES_0}] \tag{16}$$

$$\frac{d[\mathrm{S_1}]}{dt} = k_\mathrm{cat}[\mathrm{ES_0}] \tag{17}$$

$$\frac{d[\mathrm{E}]}{dt} = -k_\mathrm{f}[\mathrm{E}][\mathrm{S_0}] + k_\mathrm{r}[\mathrm{ES_0}] + k_\mathrm{cat}ES_0 \tag{18}$$

with conservation laws of substrate and enzyme, respectively, as

$$S_\mathrm{tot} = [\mathrm{S_0}]|_{t=0} = [\mathrm{S_0}] + [\mathrm{S_1}] + [\mathrm{ES_0}]\,, \tag{19}$$

$$E_\mathrm{tot} = [\mathrm{E}]|_{t=0} = [\mathrm{E}] + [\mathrm{ES_0}]\,. \tag{20}$$

The QSSA for $\mathrm{ES_0}$ gives the following simplified expressions:

$$\frac{d[\mathrm{S_0}]}{dt} = -k_\mathrm{eff}[\mathrm{E}][\mathrm{S_0}]\,, \tag{21}$$

$$\frac{d[\mathrm{S_1}]}{dt} = k_\mathrm{eff}[\mathrm{E}][\mathrm{S_0}]\,, \tag{22}$$

where

$$k_\mathrm{eff} = E_\mathrm{tot}\frac{k_\mathrm{f}k_\mathrm{cat}}{k_\mathrm{r} + k_\mathrm{cat}}\,. \tag{23}$$

If $k_\mathrm{r} \ll k_\mathrm{cat}$ the effective parameter reduces further to $k_\mathrm{eff} \approx k_\mathrm{f}$.

2. Methodology

A. Diffusion Maps. Many techniques exist for parsimoniously describing low-dimensional data sampled from high-dimensional embedding spaces, including among others Isomap (3), Local Linear Embedding (4), and Laplacian Eigenmaps (5) as well as diffusion maps (DMaps) (6), which is our preferred approach here. In this section, we first explain the algorithm in a more general way and then we illustrate how it applies to our parameter reduction problem.

Given a data set, $\mathbf{X} = \{\boldsymbol{x}_i\}_{i=1}^N$ with each $\boldsymbol{x}_i \in \mathbb{R}^m$, the first step of a DMaps algorithm is to construct a random walk on the data. This is achieved by means of an affinity matrix $\mathbf{A} \in \mathbb{R}^{N\times N}$ that characterizes the likelihood of making a transition from point $\boldsymbol{x}_i$ to $\boldsymbol{x}_j$. The entries of $\mathbf{A}$ are computed in terms of a kernel, typically the Gaussian kernel, which is defined as

$$A_{ij} = \exp\left(-\frac{\|\boldsymbol{x}_i - \boldsymbol{x}_j\|^2}{2\varepsilon}\right)\,, \tag{24}$$

where $\|\cdot\|$ denotes an appropriate norm for the observations (6, 7). In this paper, we will consider only the ℓ^2 norm. The scale parameter $\varepsilon > 0$ regulates the rate of decay of the kernel: for small values of ε, only points that are close to each other appear connected in $\mathbf{A}$, since distant points will have $A_{ij} \approx 0$.

For the purposes of discovering a low-dimensional manifold $\mathcal{M} \subset \mathbb{R}^m$ and performing dimensionality reduction, we want to recover the geometry of the manifold. If the data points $\mathbf{X}$ are non uniformly sampled on the manifold, then to compute the intrinsic dimensionality regardless of the sampling density an appropriate normalization of the affinity matrix must be performed. Define a diagonal matrix $\mathbf{P} \in \mathbb{R}^{N\times N}$ with entries

$$P_{ii} = \sum_{j=1}^N A_{ij} \tag{25}$$

and compute the normalized affinity matrix

$$\widetilde{\mathbf{A}} = \mathbf{P}^{-\alpha}\mathbf{A}\mathbf{P}^{-\alpha}, \tag{26}$$

where we choose $\alpha = 0$ if assuming uniform sampling of the data, and $\alpha = 1$ otherwise. The kernel matrix $\widetilde{\mathbf{A}}$ is renormalized again by the diagonal matrix $\mathbf{D} \in \mathbb{R}^{N\times N}$ to construct a row stochastic matrix $\mathbf{W}$:

$$\mathbf{W} = \mathbf{D}^{-1}\widetilde{\mathbf{A}} \tag{27}$$

where $\mathbf{D}$ is computed as:

$$D_{ii} = \sum_{j=1}^N \widetilde{A}_{ij}. \tag{28}$$

Computing the eigendecomposition of $\mathbf{W}$ and selecting the eigenvectors that parameterize independent directions (non-harmonic eignvectors) yields a non-linear parameterization of the original data set $\mathbf{X}$. In our work this selection was achieved by applying the algorithm suggested in (8). If the number of (non-harmonic) eigenvectors that provide this embedding is less than the number of the original dimensions of the data set, the algorithm achieves dimensionality reduction. Selecting the important eigenvectors is not as straightforward as in Principal Component Analysis (PCA), where one identifies the dimensionality of a given data set based on the energy that is captured in the leading singular vectors—often by finding a "knee" in the singular value plot, after which point a negligible fraction of the energy is contained in subsequent vectors. However, it can be achieved by sorting the eigenvectors, $\boldsymbol{\phi}_i$, based on their eigenvalues λ_i and removing eigenvectors that can be represented as functions of the previous ones (harmonics) (8). Those selected non-harmonic eigenvectors reveal the *intrinsic* geometry of a given data set $\mathbf{X}$ sampled from a manifold $\mathcal{M}$. In our work here, similar to (9), using aims to extract the *intrinsic* parameters of a model. We note that, in the presence of noise, there may be no clear threshold by which to distinguish effective parameter combinations from non-effective ones; at some point the observer must decide what is "too small to be considered," which is subjective (even when thoughtful) and therefore precarious.

Two complementary approaches can be used to extract the effective parameters of the system that are relevant for the output, resp. those that are not. The effective parameters can in principle be discovered from observations of the model output; each data point then consists of a vector of measurements from time-series of the system behavior, *e.g.*, $\boldsymbol{f}(\boldsymbol{p}_i) = [\boldsymbol{f}(t_1|\boldsymbol{p}_i), \ldots, \boldsymbol{f}(t_f|\boldsymbol{p}_i)]$, obtained for different combinations of parameter values $\boldsymbol{p}$. The affinity matrix in this context is computed as

$$A_{ij} = \exp\left(-\frac{\|\boldsymbol{f}(\boldsymbol{p}_i) - \boldsymbol{f}(\boldsymbol{p}_j)\|^2}{2\varepsilon}\right). \quad [29]$$

The obtained non-harmonic eigenvectors indicate how many parameters or combinations of parameters of the original (full) model are meaningful and give an embedding for those parameters. It is worth noting, however, that when the mapping from parameter space to the model manifold is noninvertible, different parameters may give identical model responses (output multiplicity): $\boldsymbol{f}(\boldsymbol{p}_i) = \boldsymbol{f}(\boldsymbol{p}_j)$ with $\boldsymbol{p}_i \neq \boldsymbol{p}_j$. In that case, the simple output informed kernel fails (9). To circumvent this issue Holiday holiday2019manifold proposed the use of a more informative kernel found in the Lafon's Thesis (7). In this latter case the affinity matrix is computed by taking into account both the inputs and the outputs but at different scales, as

$$A_{ij} = \exp\left(-\frac{\|\boldsymbol{p}_i - \boldsymbol{p}_j\|^2}{\varepsilon^2} - \frac{\|\boldsymbol{f}(\boldsymbol{p}_i) - \boldsymbol{f}(\boldsymbol{p}_j)\|^2}{\varepsilon^c}\right), \quad [30]$$

where $c = 4$ (for $\varepsilon < 1$) allows the disambiguation of inputs leading to the same output.

The complementary approach is used to compute the number of "non-meaningful" parameters, that do not affect the output behavior of the model. Here a data set $\mathbf{Y} = \{\boldsymbol{p}_i\}_{i=1}^N$sampled for a fixed behavior of the system (see Section Data-Driven Parameter Reduction in the main text) is needed. The identifiable effective parameters (assuming no output multiplicity) will then also be fixed; the unidentifiable combinations of parameters (those consistent with the same system behavior) may take entire continua of different values. Affinity matrix elements for pairs of points $(\boldsymbol{p}_i, \boldsymbol{p}_j)$ in this data set are computed directly in the original parameter space:

$$A_{ij} = \exp\left(-\frac{\|\boldsymbol{p}_i - \boldsymbol{p}_j\|^2}{2\varepsilon}\right). \quad [31]$$

The two approaches are complementary: the first one aims to discover the dimensionality of the "meaningful" effective parameters, while the second approach addresses the dimensionality of the ones "non-meaningful" for the output. The total number of meaningful and non-meaningful parameters should then add up to the number of the original parameters of the model.

B. Nyström Extension. The Nyström extension is a technique for finding numerical approximations to eigenfunction problems of the form (10):

$$\int_a^b W(x_j, x_i)\phi(x_i)\, dx_i = \lambda\phi(x_j) \quad [32]$$

In our framework, Nyström is used as a simple extension tool to generate the DMaps coordinates $\boldsymbol{\phi}_{new}$ for new, previously unseen sample points $\boldsymbol{x}_{new} \notin \mathbf{X}$. This interpolation scheme, $f : \boldsymbol{x}_{new} \mapsto \boldsymbol{\phi}_{new}$, requires recomputing the kernel that was used during the dimensionality reduction step (and applying the same normalizations) discussed in Section A. The Nyström extension formula reads:

$$\phi_\beta(\boldsymbol{x}_{new}) = \frac{1}{\lambda_\beta}\sum_{i=1}^{N} W(\boldsymbol{x}_{new}, \boldsymbol{x}_i)\phi_\beta(x_i) \quad [33]$$

where $\phi_\beta(x_i)$ is the i-th component of the β-th eigenvector ($\boldsymbol{\phi}_\beta$) and λ_β is the β-th eigenvalue.

C. Double Diffusion Maps and their Geometric Harmonics. Geometric harmonics (GH) (7, 11) is a scheme based on the Nyström method (12), *traditionally* used for extending a function f defined on a data set $\mathbf{X}$ sampled from a manifold $\mathcal{M}$ for $\boldsymbol{x}_{new} \notin \mathbf{X}$.

In our case, we mostly aim to extend functions defined *not in the ambient space coordinates*, but on the discovered latent-reduced coordinates $\boldsymbol{\phi}$. Therefore, GH needs to be computed on only these few "governing" coordinates $\boldsymbol{\phi}$. Before we explain the algorithm, it is important to make clear why, without this additional step, the mapping from the reduced coordinates to any function defined on the ambient space would not be possible. We remind the reader that, during the first round of DMaps on $\mathbf{X}$, we discovered the intrinsic dimensionality along with the corresponding set of a few variables $\boldsymbol{\phi}$. These new variables were obtained as eigenvectors of an eigendecomposition. Of course, they were not the *only* eigenvectors computed; but they were necessary and sufficient eigenvectors to achieve the dimensionality reduction. All the other harmonic eigenvectors were "discarded" (8). Discarding those eigenvectors and keeping only the non-harmonic ones achieves the desired dimensionality reduction (and the corresponding reduced embedding) but is unable to accurately approximate a function on the manifold based on the reduced coordinates only: GH with only the governing eigenvectors gives a (possibly badly) truncated reconstruction of the function. If, however, we perform again DMaps on these few governing DMaps coordinates $\boldsymbol{\phi}$ ("Double DMaps"), and compute a new full set of eigenvectors, $\boldsymbol{\Psi}$, we obtain a full basis for expressing functions *on the reduced manifold* - and therefore, functions on the original data. We mention again that it is not necessary to use the *absolute minimal* number of Dmaps eigenvectors that parameterize the manifold; more than the minimal will, in principle, also work well for function reconstruction. Differentiating the approximated (via Double DMaps GH) function with respect to the governing DMaps coordinates (either symbolically or via automatic differentiation) is what will allow us to test, with the help of the Inverse Function Theorem (Section E), the explainability of these coordinates in terms of physical parameters. In addition, it allows us to perform parameter estimation for new unseen behaviors.

As in "single" DMaps, the first step for GH is to compute an affinity matrix:

$$A_{ij} = \exp\left(-\frac{\|\boldsymbol{\phi}_i - \boldsymbol{\phi}_j\|^2}{2\varepsilon}\right) \qquad [34]$$

Since it is symmetric and positive semidefinite, this matrix $\mathbf{A}$ has a set of orthonormal vectors $\psi_0, \psi_1, \ldots, \psi_{N-1}$ and non-negative eigenvalues $(\sigma_0 \geq \sigma_1 \geq \cdots \geq \sigma_{N-1} \geq 0)$ (13). Those eigenvectors are used as a basis set onto which we project and subsequently extend the function of interest f. More precisely, for $\delta > 0$ we consider the set of truncated eigenvalues $S_\delta = \{\alpha : \sigma_\alpha > \delta\sigma_0\}$. In this truncated set we project f evaluated in some scatter points:

$$f \mapsto P_\delta f = \sum_{\alpha \in S_\delta} \langle f, \psi_\alpha \rangle \psi_\alpha \,, \qquad [35]$$

where $\langle \cdot, \cdot \rangle$ is the inner product.The extension of f for $\boldsymbol{\phi}_{new} \notin \Phi$ (or $\boldsymbol{x}_{new} \notin \mathbf{X}$) is defined as:

$$(Ef)(\boldsymbol{\phi}_{new}) = \sum_{\alpha \in S_\delta} \langle f, \boldsymbol{\psi}_\alpha \rangle \Psi_\alpha(\boldsymbol{\phi}_{new}) \,, \qquad [36]$$

where

$$\Psi_\alpha(\boldsymbol{\phi}_{new}) = \sigma_\alpha^{-1} \sum_{i=1}^{N} A(\boldsymbol{\phi}_{new}, \boldsymbol{\phi}_i) \psi_\alpha(\phi_i) \qquad [37]$$

and $\psi_a(\phi_i)$ is the ith component of the DMaps eigenvector $\boldsymbol{\psi}_\alpha$. The function Ψ_α are the GH we use. It is worth noting that using a truncated set S_δ is important to circumvent the numerical instabilities that will arise in Equation 37 when $\sigma_i \to 0$.

Beyond enabling the extension of a function defined on $\mathbf{X}$ (or $\boldsymbol{\Phi}$) GH can be used to approximate the gradient of the function in terms of the original variables (or the variables). Symbolic differentiation of Equation 37 gives a closed form expression of the gradient of f in term of the independent variables. Having this capability in our "toolkit" allows us to perform scientific computation without relying on, say, a finite difference scheme:

$$\mathbf{D}\Psi_\alpha(\boldsymbol{\phi}_{new}) = \sigma_\alpha^{-1} \sum_{i=1}^{N} -\frac{(\boldsymbol{\phi}_{new} - \boldsymbol{\phi}_i)}{\varepsilon} A(\boldsymbol{\phi}_{new}, \boldsymbol{\phi}_i) \psi_\alpha(\phi_i) \,. \qquad [38]$$

We could also computing the gradient of f with automatic differentiation of Equations 36 and 37.

D. Choosing Base Parameter Values, Representative Initial Conditions, and More. The computations we report are obtained by sampling the model response in a finite neighborhood of a single "base point" in parameter space, and for a single given set of "reference" initial conditions. Generically, in simulating an n-dimensional nonconservative dynamical system, if more than one attractors exist, their basins of attraction are also n-dimensional; perturbing a random initial condition within one basin will not affect the ultimate behavior, which will eventually approach the same attractor. The boundaries separating different basins are co-dimensional sets (so $n-1$ dimensional), so the points on them are much more "rare" (a set of measure zero) compared to points in any basin. In the same spirit, the reference initial condition choice will not generically affect the dimensionality of the model manifold, and therefore our estimation of the number of effective parameters. The initial conditions in whose neighborhood the model manifold dimension actually changes, we expect to be non-generic (also measure zero, *i.e.*, of lower dimension than the generic ones). In that sense, choosing a reference initial condition randomly should be representative.

Selecting the base point in parameter space, however, requires more discussion. Away from regimes where the QSSA leads to lower-dimensional behavior, any base point in a finite neighborhood will lead to the same model manifold dimensionality. Yet this would be different from the model manifold dimensionality observed at base points in regimes where the QSSA assumptions holds. So, while the precise base point is not important, *the parameter space regime* in which it is chosen (and in which the model manifold dimensionality remains the same) does matter. Characterizing these different regimes, and their relations to each other, the model manifold and its boundaries, constitutes part of the Model Boundary Approximation Method (14). For an illustrative study of transitions from parameter regimes with one model manifold dimensionality to parameter regimes with a different model manifold dimensionality see also (9).

Finally, it is worth mentioning that "the scale of the observer" (the units in which the measurements are recorded, and the time intervals allowed to elapse between successive measurements) may also very much affect the numerical determination of the dimensionality of the response. If, for example, the time intervals in our time series measurements are extremely small, it will appear that the solution simply does not (appreciably numerically) change, even when the base parameters are changing.

E. Explainability: Inverse Function Theorem. Consider a linear system of n equations in n variables, which may be written in full as

$$\begin{matrix} a_{11}x_1 + \cdots + a_{1n}x_n = y_1 \\ \vdots \quad \ddots \quad \vdots \quad \vdots \\ a_{n1}x_1 + \cdots + a_{nn}x_n = y_n \end{matrix} \qquad [39]$$

or succinctly as the matrix equation $\mathbf{A}\boldsymbol{x} = \boldsymbol{y}$. This system has a unique solution $\boldsymbol{x}_\star = \mathbf{A}^{-1}\boldsymbol{y}$ if and only if the matrix $\mathbf{A}$ is invertible. For nonlinear systems of the form

$$\begin{matrix} f_1(x_1, \ldots, x_n) = y_1 \\ \vdots \quad \ddots \quad \vdots \quad \vdots \\ f_n(x_1, \ldots, x_n) = y_n \end{matrix} \quad , \qquad [40]$$

however, we are generally limited to techniques that provide local information about possible solutions $\boldsymbol{x}$ to the system $f(\boldsymbol{x}) = \boldsymbol{y}$. Suppose that $\boldsymbol{x} \in \mathbb{R}^n$ is such a solution and $f : \mathbb{R}^n \to \mathbb{R}^n$ is a differentiable function. The Inverse Function Theorem (15) states that, if the Jacobian matrix

$$\mathbf{J}f(\boldsymbol{x}) = \begin{bmatrix} \frac{\partial f_1}{\partial x_1} & \cdots & \frac{\partial f_1}{\partial x_n} \\ \vdots & \ddots & \vdots \\ \frac{\partial f_n}{\partial x_1} & \cdots & \frac{\partial f_n}{\partial x_n} \end{bmatrix} \qquad [41]$$

is invertible, then we can find neighborhoods of $\boldsymbol{x}$ and $\boldsymbol{y}$ for which an inverse function f^{-1} : exists that specifies a unique (local) solution $\boldsymbol{x}_\star$ for any $\boldsymbol{y}_\star$ sufficiently close to $\boldsymbol{y}$.

For data-driven applications, we are interested in demonstrating that there exists a globally one-to-one mapping between a set of inputs $\{\boldsymbol{x}_i\}$ and a set of outputs $\{\boldsymbol{y}_i\}$, without any knowledge of an analytical expression for the relationship between the two. If we can compute or approximate all first-order partial derivatives in Equation 41, then we can assess the invertibility of f on the basis of the Jacobian's determinant at each input. A square matrix is invertible if and only if its determinant is nonzero, so finding that $\det \mathbf{J}f(\boldsymbol{x}_i)$ takes values of a single sign on our input set suggests that the input-output mapping is a one-to-one relationship. However, we must also consider that it is possible for a function to be locally invertible everywhere but not globally invertible. Thus, we must also check the data to ensure that the only pairs of observations with similar outputs, $\|\boldsymbol{y}_i - \boldsymbol{y}_j\| \approx 0$ also have similar inputs, $\|\boldsymbol{x}_i - \boldsymbol{x}_j\| \approx 0$.

F. Determining Level Sets in Practice. Consider a dynamic model involving m physical parameters. We observe n output quantities of this model at a fixed parameter vector $\boldsymbol{p}$:

$$\boldsymbol{F} : \mathbb{R}^m \to \mathbb{R}^n : \boldsymbol{p} \mapsto \begin{bmatrix} F_1(p_1, \ldots, p_m) \\ \vdots \\ F_n(p_1, \ldots, p_m) \end{bmatrix} . \qquad [42]$$

Suppose, however, that the dependence of $\boldsymbol{F}$ on $\boldsymbol{p}$ can be reduced to $d < m$ effective parameters,

$$\boldsymbol{q} = \begin{bmatrix} \phi_1(p_1, \ldots, p_m) \\ \vdots \\ \phi_d(p_1, \ldots, p_m) \end{bmatrix} , \qquad [43]$$

such that the underlying behavior $f : \mathbb{R}^d \to \mathbb{R}^n$ satisfies $\boldsymbol{F}(\boldsymbol{p}) = \boldsymbol{f}(\boldsymbol{q})$. Applying the multivariate chain rule to $\boldsymbol{F} = \boldsymbol{f} \circ \boldsymbol{\phi}$, we have

$$JF(\boldsymbol{p}) = [Jf(\boldsymbol{q})]\,[J\phi(\boldsymbol{p})] \in \mathbb{R}^{n\times m} , \qquad [44]$$

and, since $\phi : \mathbb{R}^m \to \mathbb{R}^d$, it follows that $\mathrm{rk}[JF(\boldsymbol{p})] \leq \mathrm{rk}[J\phi(\boldsymbol{p})] \leq d$.

Let $\boldsymbol{p} \in \mathbb{R}^m$ be a vector of physical parameter values in which we have some interest and let $\boldsymbol{q} = \phi(\boldsymbol{p})$ and $\boldsymbol{y} = f(\boldsymbol{q}) = F(\boldsymbol{p})$ be the corresponding effective parameters and outputs, respectively. We are interested in the $(m-d)$-dimensional level set of physical parameter values that produce the same output observations:

$$\mathcal{M} = \{\boldsymbol{v} \in \mathbb{R}^m \mid F(\boldsymbol{v}) = \boldsymbol{y}\}\,. \qquad [45]$$

The tangent space to this manifold at $\boldsymbol{p}$ is given by the nullspace of the Jacobian:

$$T_{\boldsymbol{p}}\mathcal{M} = \mathcal{N}(\mathbf{J}\boldsymbol{F}(\boldsymbol{p})) = \{\boldsymbol{v} \in \mathbb{R}^m \mid [\mathbf{J}\boldsymbol{F}(\boldsymbol{p})]\boldsymbol{v} = \mathbf{0}\}\,, \qquad [46]$$

which consists of the directions along which a local linear approximation of the output predicts no change. Given the Jacobian matrix $\mathbf{J}\boldsymbol{F}(\boldsymbol{p})$ (also called the Sensitivity Matrix (16)) at a point $\boldsymbol{p}$, we can obtain a basis for its nullspace from a Singular Value Decomposition (SVD) (17):

$$\mathbf{J}F(\boldsymbol{p}) = \mathbf{U}\boldsymbol{\Sigma}\mathbf{V}^\top \in \mathbb{R}^{n\times m}\,, \qquad [47]$$

in which $\mathbf{U} \in \mathbb{R}^{n\times n}$ and $\mathbf{V} \in \mathbb{R}^{m\times m}$ are orthogonal matrices and $\boldsymbol{\Sigma} \in \mathbb{R}^{n\times m}$ has all zero entries except on the main diagonal, where $\Sigma_{ii} = \sigma_i \geq 0$. The nullspace $\mathcal{N}(\mathbf{J}F(\boldsymbol{p}))$ is spanned by the columns of $\mathbf{V}$ that correspond to those singular values σ_i that equal zero. Alternatively, one could perform an eigendecomposition of the *sensitivity Fisher Information matrix*, which is defined in (16) as $[\mathbf{J}F(\boldsymbol{p})]^\top \mathbf{J}F(\boldsymbol{p}) \in \mathbb{R}^{m\times m}$ and corresponds to the expected value of the Fisher Information Matrix in the case of standard Gaussian measurement error.

This approach provides the tangent space only at the point $\boldsymbol{p}$ for which we compute the Jacobian. The data-driven methods proposed in this paper provide much more than a local tangent space: the entire (global over our data) level set manifold; an orthogonal set of coordinates on it; as well as a completion of this set, through our redundant parameter combinations, to coordinates orthogonal over the entire parameter space.

G. The Conformal Autoencoder Network. For the calculations included in this paper, all sub-networks of the Y-shaped conformal autoencoder network had the same specifications: five fully connected linear layers with 20 neurons each; the first four layers have a $\tanh(t)$ activation function and the last one is linear. Algorithm 1 illustrates the training scheme used for our Conformal Autoencoder.

In the example presented in this work, we specifically used `ADAM` as the optimizer. The optimization process is heuristic: note that one "epoch" consists of two optimization steps, one updating the $(\mathrm{NN}_1, \mathrm{NN}_2)$ network which is an autoencoder, and one updating the $(\mathrm{NN}_1, \mathrm{NN}_3)$ network. This algorithm does not include the additional step of training NN_4 of Figure 9 of the main text. Alternative formulations of the training protocol are, of course, possible. The structure of the architecture is more generally applicable, beyond the specific choices made here, and optimizing it is a topic of current research.

In Figure S1, we illustrate how our Y-shaped conformal autoencoder will look for the MSP model discussed in Section A. In this case, we can directly map from the new full input vector to *both* (a) the effective parameters of the Autoencoder ν_1, ν_2, ν_3 and (b) from those latent descriptors to the estimated behavior. Alternatively, the path from $\boldsymbol{\nu}$ to the behavior can be implemented by using GH from the effective parameters of the Autoencoder to the DMaps coordinates and then from the DMaps coordinates with our Double DMaps GH scheme to the behaviors.

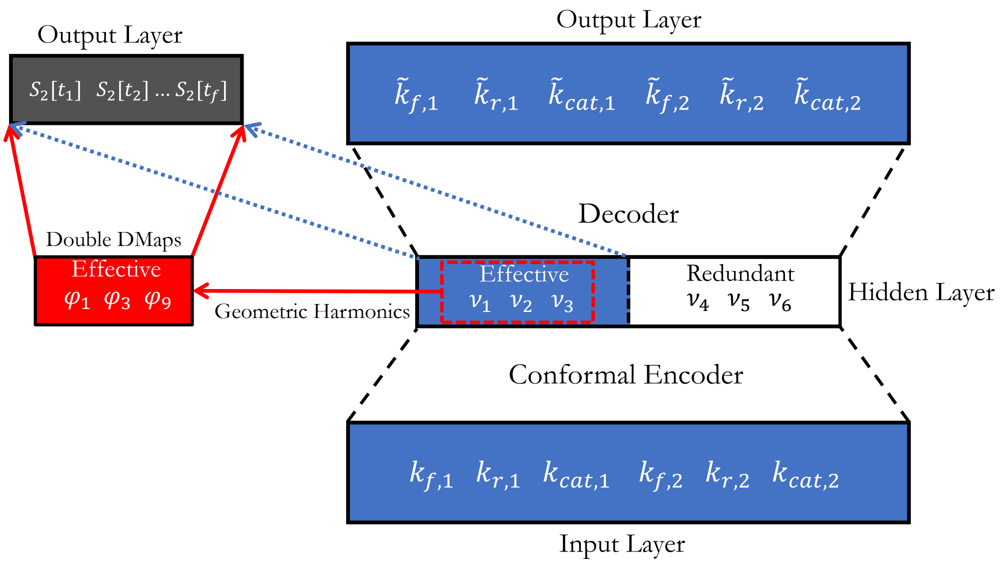

Fig. S1. A schematic of the Y-shaped Conformal Autoencoder for the MSP example combined with our manifold learning scheme allows to make predictions for systems' behaviors given new unseen full input vector $\boldsymbol{p}$.

Algorithm 1 Conformal Autoencoder Training: we used a hyperparameter value $\alpha = 33$ to scale the relative importance of the orthogonality relation; $\boldsymbol{S}$ is the vector of true output behaviors; and $\tilde{\boldsymbol{S}}$ is the estimate from the network.

Input:Data $k_{\mathrm{f}}, k_{\mathrm{r}}, k_{\mathrm{cat}}$ and output behaviors $\mathbf{S}$.
Output: The weights of the three neural networks $\{\theta_{NN_1}, \theta_{NN_2}, \theta_{NN_3}\}$.
For $t = 1, 2, ..., T$

1. Predict:
$$\big(\nu_1, \nu_2, \nu_3\big) = \mathrm{NN}_1(k_{\mathrm{f}}, k_{\mathrm{r}}, k_{\mathrm{cat}})$$
2. $$\big(\tilde{k}_{\mathrm{f}}, \tilde{k}_{\mathrm{r}}, \tilde{k}_{\mathrm{cat}}\big) = \mathrm{NN}_2\big(\nu_1, \nu_2, \nu_3\big)$$
3. Compute Autoencoder and Conformality Losses:
$$L_1 = \mathrm{MSE}(\tilde{\boldsymbol{k}}, \boldsymbol{k}) + \alpha \sum_{\{(i,j):j>i\}} \mathrm{MSE}(\langle \boldsymbol{d\nu}_i, \boldsymbol{d\nu}_j \rangle, 0)$$
4. Update Weights (here we just show gradient descent) :
$$\theta_{\mathrm{NN}_1} -= \eta_1 \boldsymbol{\nabla}_{\theta_{\mathrm{NN}_1}} L_1$$
$$\theta_{\mathrm{NN}_2} -= \eta_2 \boldsymbol{\nabla}_{\theta_{\mathrm{NN}_2}} L_1$$
5. $$\big(\nu_1, \nu_2, \nu_3\big) = \mathrm{NN}_1(k_{\mathrm{f}}, k_{\mathrm{r}}, k_{\mathrm{cat}})$$
6. $$\tilde{\boldsymbol{S}} = \mathrm{NN}_3\big(\nu_1\big)$$
7. Compute Behavior Estimator Loss:
$$L_2 = \mathrm{MSE}(\tilde{\boldsymbol{S}}, \boldsymbol{S})$$
8. Update Weights (here we just show gradient descent):
$$\theta_{\mathrm{NN}_1} -= \eta_1 \boldsymbol{\nabla}_{\theta_{\mathrm{NN}_1}} L_2$$
$$\theta_{\mathrm{NN}_3} -= \eta_3 \boldsymbol{\nabla}_{\theta_{\mathrm{NN}_3}} L_2$$

H. Jointly Smooth Functions. Jointly Smooth Functions (JSFs) (18) provide an alternative kernel-based pathway to obtaining effective parameters. The key idea for constructing JSFs between several (say, K) data sets, arising from different observations of the same phenomenon, is to define function spaces on all K data sets separately, through eigenvectors of kernels that we will describe, and then use a singular value decomposition to find the "common" functions across these spaces. For details, see (18). In our case, we have two data sets: the set of input/parameter settings for each simulation, and the set of output measurements for that input, and we will have to perform two eigendecompositions and a subsequent SVD. The "common" functions between input and output correspond to our effective parameters (meaningful parameter combinations, that affect the output); the "uncommon" functions between input and output correspond to our redundant parameter combinations, that do not affect the output.

Algorithm 2 Jointly Smooth Functions from K sets of observations.

Input: K sets $\left\{\boldsymbol{x}_i^{(1)}, \boldsymbol{x}_i^{(2)}, \ldots \boldsymbol{x}_i^{(K)}\right\}_{i=1}^{N}$ where $\boldsymbol{x}_i^{(k)} \in \mathbb{R}^{d_k}$.
Output: M jointly smooth functions $\{\boldsymbol{f}_m \in \mathbb{R}^N\}_{m=1}^{M}$.

1. For each observation set $\left\{\boldsymbol{x}_i^{(k)}\right\}_{i=1}^{N}$ compute the kernel:

$$K_k(i,j) = \exp\left(-\frac{\left\|\boldsymbol{x}_i^{(k)} - \boldsymbol{x}_j^{(k)}\right\|^2}{2\sigma_k^2}\right)$$

2. Compute $\mathbf{W}_k \in \mathbb{R}^{N\times d}$, the first d eigenvectors of $\mathbf{K}_k$.
3. Set $\mathbf{W} =: [\mathbf{W}_1, \mathbf{W}_2, \ldots, \mathbf{W}_K] \in \mathbb{R}^{N\times Kd}$
4. Compute the SVD decomposition: $\mathbf{W} = \mathbf{U}\Sigma\mathbf{V}^T$
5. Set $\boldsymbol{f}_m$ to be the mth column of $\mathbf{U}$.

An Illustrative JSF Example We illustrate through a toy example how the JSF algorithm discovers directions that are common between two data sets as well as directions that are "uncommon" between them, *i.e.*, there are specific two the one or the other data set. For our application, the first data set consists of the parameter values and the second data set consists of the output measurements observed for these parameter values. For us the "common" directions between parameters and output observations correspond to our meaningful effective parameters; and the directions that are "uncommon" between parameters and output observations correspond to our redundant parameter combinations. Consider the random variable triplet $(a_i, b_i, c_i) \sim U\,[-0.5, 0.5]^3$, iid and uniformly distributed. Define the "common" direction as $z_i = a_i + b_i^2$, and consider the first set of observations to be $\boldsymbol{x}_i = (a_i, b_i)$. The second set of observations is arranged on a spiral in $\mathbb{R}^2$ given by

$$\boldsymbol{y}_i = \begin{bmatrix} \left(\frac{c_i}{2} + \frac{z_i}{4} + \frac{1}{3}\right)\cos(2\pi c_i) \\ \left(\frac{c_i}{2} + \frac{z_i}{4} + \frac{1}{3}\right)\sin(2\pi c_i) \end{bmatrix}. \quad [48]$$

The two sets are shown in Figure S2, with the common direction z shown in color.

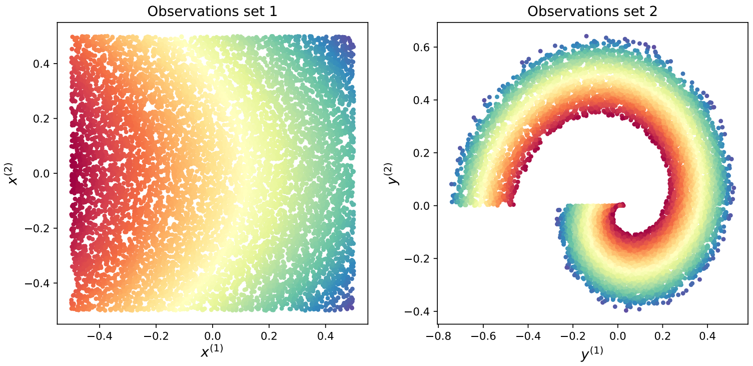

Fig. S2. Two sets of measurements involving a common variable and "set-specific" uncommon variables, described in the text. Color indicates the "common direction" between the two data sets.

Using Algorithm 2 to detect the variable that is "common" (jointly smooth), we then try to determine what is "uncommon" (sensor-specific) across our data-sets. In the example shown in Figure S2: how do we obtain a parameterization along the

arclength of the spiral (uncommon between the data sets), as opposed to the parameterization across its width (common between the data sets)?

Computationally identifying uncommon directions in the JSF framework can be performed as follows. After computing a set of JSFs $\boldsymbol{f}_{\text{JSF}}$ between the two data sets to obtain basis functions for the common subspace, we consider only one of the data sets (*e.g.*, the spiral) and remove all of the common (jointly smooth) functions from the vector space spanned by the kernel eigenfunctions we computed in Algorithm 2. Because the kernel eigenfunctions parameterize all functions on the manifold, what remains after removing common directions are functions that we expect to parameterize the uncommon directions. One issue with this approach is that we typically do not obtain enough JSFs to accurately span a large number of functions in the common directions. This would imply that removing only the small number of JSFs leaves too many common directions in the full space, and the uncommon eigendirections are still mixed with the common ones. To alleviate the problem of factoring out too few JSFs, in Algorithm 4, we perform a different "double" process: by applying the Algorithm (6) to the few detected JSFs, we obtain a large number of smooth functions that span a larger portion of the function space on the common manifold. This larger number of common functions is then used to further enhance factoring out (removing the influence of) the common directions from the full space (Algorithm 4, Step 2). When we apply Algorithm 4 to the spiral data, we obtain the results shown in Figure S3. Figure S4 compares the uncommon directions with the redundant coordinates identified by our conformal autoencoder.

Algorithm 3 Obtaining uncommon directions in a function space.

Input: Full function space $\boldsymbol{f}_{\text{full}} \in \mathbb{R}^{N\times K}$, subspace to remove $\boldsymbol{f}_{\text{remove}} \in \mathbb{R}^{N\times R}$.
Output: Full space $\boldsymbol{f}_{\text{uncommon}} \in \mathbb{R}^{N\times K}$, with all functions only containing information in the uncommon directions.
Algorithm:

1. Compute the projection of all functions on the functions to remove:
$$c := \boldsymbol{f}_{\text{full}}^T \boldsymbol{f}_{\text{remove}} \in \mathbb{R}^{K\times R}.$$
2. For $i = 1, \ldots, K$, select the i-th row of c, transpose, and multiply with the subspace to remove:
$$\mathbb{R}^N \ni \boldsymbol{r}_i = \boldsymbol{f}_{\text{remove}} \underbrace{c_i^T}_{\in \mathbb{R}^R}.$$
3. For every function $\boldsymbol{f}_{\text{full},i} \in \mathbb{R}^N$, remove the contribution of all functions in the subspace:
$$\boldsymbol{f}_{\text{uncommon},i} = \boldsymbol{f}_{\text{full},i} - \boldsymbol{r}_i\,.$$

Algorithm 4 Obtaining uncommon directions on a manifold.

Input: Jointly smooth functions $\boldsymbol{f}_{\text{JSF}} \in \mathbb{R}^{N\times M}$, kernel eigenvectors $\boldsymbol{f}_{\text{kernel}} \in \mathbb{R}^{N\times K}$.
Output: Uncommon directions $\boldsymbol{f}_{\text{uncommon}} \in \mathbb{R}^{N\times M}$, with all functions only containing information in the uncommon directions.
Algorithm:

1. Apply DMaps to $\boldsymbol{f}_{\text{JSF}}$ to obtain a list of R smooth functions $\Phi := (\phi_1, \ldots, \phi_R) \in \mathbb{R}^{N\times R}$, $R \gg M$, on the common directions, sorted by smoothness (DMaps eigenvalue).
2. Apply algorithm 3 to the full space $\boldsymbol{f}_{\text{kernel}}$, removing the subspace Φ, to obtain $\boldsymbol{f}_{\text{kernel, uncommon}}$.
3. Apply the JSF algorithm to the following two data sets: (a) $\boldsymbol{f}_{\text{kernel, uncommon}}$ and (b) the observations $\boldsymbol{y}$ (the ones used to create $\boldsymbol{f}_{\text{kernel}}$). This creates a list of M functions $\boldsymbol{f}_{\text{uncommon}} \in \mathbb{R}^{N\times M}$ that are "jointly smooth" between the uncommon functions obtained in step 2 and the original coordinates of the manifold, $\boldsymbol{y}$.

JSF Computations for Our Second Example. For our second example (Section On the Parameter Combinations that do not Matter in the main text), we computed through Algorithm 4 also the redundant parameter combinations. The "uncommon" JSFs thus discovered for our second example are colored with the Conformal Autoencoders' redundant coordinates. The figures support visually the one-to-one relationship between the two descriptions.

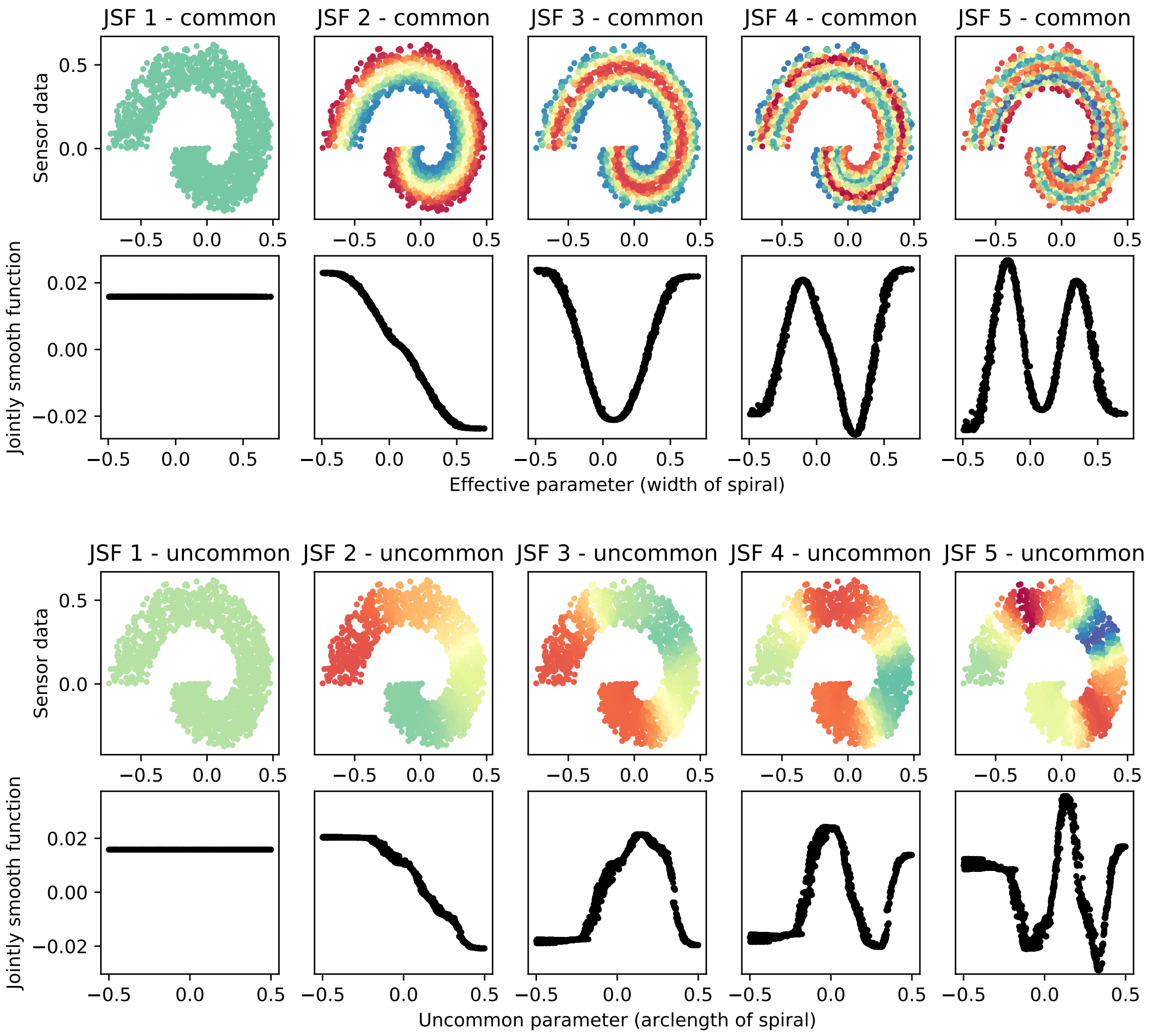

Fig. S3. Common and uncommon functions extracted with the (extension of the) JSF algorithm on the spiral data set. Upon inspection, they can be rationalized as harmonics along the width (common) vs. harmonics along the arclength (the uncommon) directions.

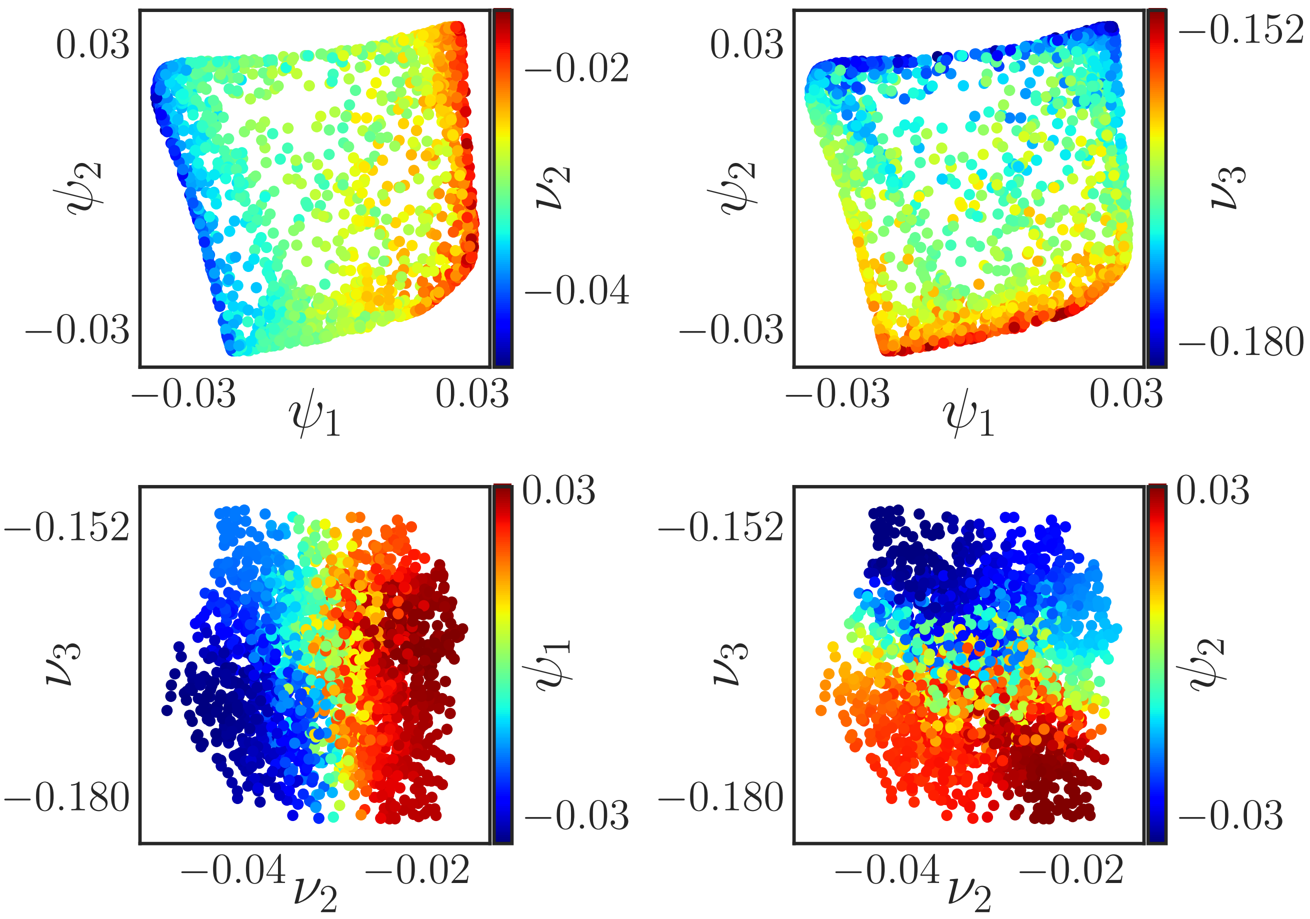

Fig. S4. [Top] the uncommon (between parameters and output) JSFs, colored by the two redundant coordinates of the Conformal Autoencoder. [Bottom] the redundant coordinates of the Conformal Autoencoder, colored by the two uncommon JSFs.

3. Another Base Parameter Value Set for our Toy Example

For the second toy example we discussed in Section On the Parameter Combination that do not Matter in the main text we also show what we find, with our scheme, around a different, even "simpler" base value, $\boldsymbol{k}_1 = (k_\mathrm{f}, k_\mathrm{r}, k_\mathrm{cat}) = (0.71, 19, 6700)$. We follow the same algorithmic procedure, and compare our data driven effective parameter, obtained from the output informed DMaps, with the theoretical effective parameter based on QSSA. In this regime since $k_\mathrm{cat} \gg k_\mathrm{r}$ the effective parameter based on the QSSA reads:

$$k_\mathrm{eff} = E_\mathrm{tot}\frac{k_\mathrm{f} k_\mathrm{cat}}{k_\mathrm{r} + k_\mathrm{cat}} \simeq E_\mathrm{tot} k_\mathrm{f} \quad [49]$$

In this parameter regime, Figure S5 demonstrates that our manifold learning approach discovers a single data driven effective input, ϕ_1, the first nontrivial eigenvector of our output-informed Dmap computation. We confirm that this ϕ_1 is one-to-one with the analytically (QSSA) obtainable effective parameter k_eff. In this regime, k_eff is practically indistinguishable from the k_f input, and the level sets of ϕ_1 (implicitly, the levels set of k_eff and k_f) are simply planes orthogonal to the k_f axis (parallel to the k_cat and k_r axes in full input space).

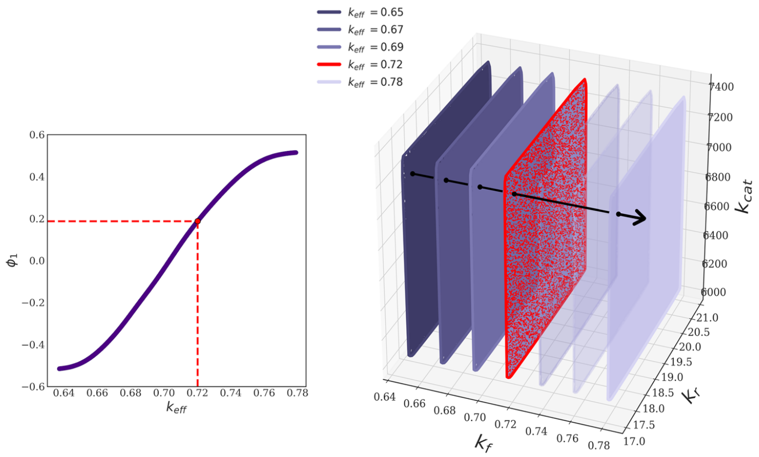

Fig. S5. [Left] the data-driven coordinate ϕ is one-to-one with the effective parameter k_eff. [Right] levels sets of constant behaviors, the level sets are parallel to k_cat, k_r. The red level set corresponds to the parameter combinations k_cat, k_r that give the behavior indicated with red point.

4. Errors Fitting GH and NN

In this section, we report the errors computed for the different regression schemed mentioned in the main paper. The mean absolute percentage error for 3,000 tests points of the forward map, $f : \Phi \to K$, is reported for the interpolation schemes (Double DMaps GH and the Neural Network). Table S1. In Table S2, the mean absolute percentage error for the *inverse* map,

Method	κ_1	κ_2	π
Double DMaps	3.2×10^{-3} %	1.5×10^{-4} %	6.2×10^{-3} %
Neural Network	3.2×10^{-2} %	4.0×10^{-2} %	3.4×10^{-2} %

Table S1. Mean absolute percentage error for the GH and the neural network interpolation scheme $f : \Phi \to K$.

$f^{-1} : K \to \Phi$ computed with the Neural is reported for the three DMaps coordinates. The mean absolute percent error for the

Method	ϕ_1	ϕ_3	ϕ_9
Neural Network	2.9×10^{-2} %	5.2×10^{-2} %	5.4×10^{-2} %

Table S2. Mean absolute percentage error with the neural network interpolation scheme $f^{-1} : K \to \Phi$.

prediction of effective parameters given unseen behaviors (Section Behavior Estimation in the main text) is shown in Table S3. It is worth mentioning that the pre-trained Double DMaps GH for the mapping $f : \Phi \to K$ used for the Nyström formula was also used here. The reason we report also here the error is because of the use of the Nyström extension formula for the restriction of the output observations to the reduced DMaps coordinates.

Method	κ_1	κ_2	π
Double DMaps	3.0×10^{-3} %	2.6×10^{-4} %	6.6×10^{-3} %

Table S3. Mean absolute percentage error with the Double DMaps scheme for the prediction of effective parameters κ for values of unseen behaviors.

5. Compartmental Models: A Textbook Nonidentifiability Example

Compartmental models describe the exchange of matter or energy between different states (19). This makes them suitable to applications in ecology, kinetics, separation processes and more (20, 21). Such models consist of a system of coupled, first-order ordinary differential equations (ODEs). A *linear* compartmental model with n compartments can be written (19) in the form

$$\begin{aligned} \boldsymbol{y}(t,\boldsymbol{p}) &= C(\boldsymbol{p})\,\boldsymbol{x}(t,\boldsymbol{p})\,, \\ \frac{\partial \boldsymbol{x}}{\partial t} &= A(\boldsymbol{p})\,\boldsymbol{x}(t,\boldsymbol{p}) + B(\boldsymbol{p})\,\boldsymbol{u}(t)\,, \end{aligned} \qquad [50]$$

where A, B, and C are known matrix functions of a parameter vector $\boldsymbol{p} \in \mathbb{R}^d$; $\boldsymbol{u}(t)$ defines the input to the system; $\boldsymbol{x} \in \mathbb{R}^n$ are the internal system states; and $\boldsymbol{y} \in \mathbb{R}^m$ is a vector of observed quantities.

We borrow a textbook two-compartment model, illustrated in Figure S6, for which we observe the scalar quantity $y(t) = x_1(t)$ given

$$\begin{aligned} A &= \begin{bmatrix} -(p_{10}+p_{12}) & p_{21} \\ p_{12} & -(p_{20}+p_{21}) \end{bmatrix}, & B &= \begin{bmatrix} 1 \\ 0 \end{bmatrix}, \\ C &= \begin{bmatrix} 1 & 0 \end{bmatrix}, & u(t) &= \delta(t)\,, \end{aligned} \qquad [51]$$

where $\delta(\cdot)$ represents a unit impulse at initial time $t = 0$. Cole (19) demonstrates that this model is structurally nonidentifiable: its four parameters can be reduced to a set of three:

$$\boldsymbol{\beta} = \begin{bmatrix} \beta_1 \\ \beta_2 \\ \beta_3 \end{bmatrix} = \begin{bmatrix} p_{10}+p_{12} \\ p_{20}+p_{21} \\ p_{12}\,p_{21} \end{bmatrix}. \qquad [52]$$

We select as our base point $\tilde{\boldsymbol{p}} = (1, 1, 1, 1)$ and take our output observation function to be

$$\boldsymbol{y}(\boldsymbol{p}) = \begin{bmatrix} x_1(0.5,\boldsymbol{p}) & x_1(1.0,\boldsymbol{p}) & \cdots & x_1(5.0,\boldsymbol{p}) \end{bmatrix}^\top \in \mathbb{R}^{10}\,, \qquad [53]$$

subject to fixed initial conditions $\boldsymbol{x}(0) = \begin{bmatrix} 1 & 0 \end{bmatrix}^\top$ (resulting from the impulse input u). The dynamic behavior starting at this base point, $\tilde{\boldsymbol{y}} = \boldsymbol{y}(\tilde{\boldsymbol{p}})$, is illustrated in Figure S7. We generate $N = 5000$ parameter vectors $\{\boldsymbol{p}_i\}_{i=1}^N$ by independently and uniformly perturbing each component within $\pm 10\%$ of its base value and record the corresponding output response history vectors $\{\boldsymbol{y}_i\}_{i=1}^N$. We used these first 5000 parameter settings to train our GH and NN models, while similarly generating an additional $N' = 500$ as a test set, $\{\boldsymbol{p}'_i\}_{i=1}^{N'}$.

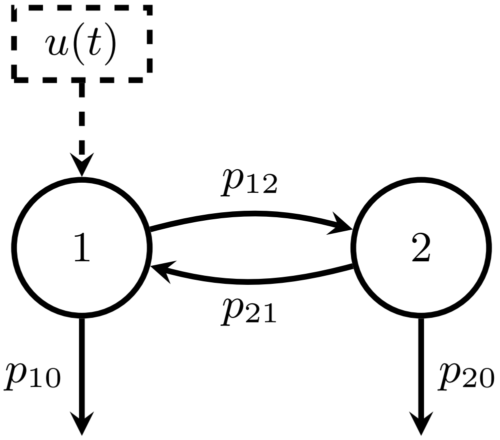

Fig. S6. Schematic of a compartmental model with four parameters, specifying the rates at which material is exchanged (p_{12} and p_{21}) between two compartments and flows out of the system (p_{10} and p_{20}). In the case of Equation (50), an impulse input $u(t)$ is initially supplied to the first compartment, the contents of which constitute our observations.

We computed diffusion maps (DMaps) on the training output vectors and found that $(\phi_1, \phi_5, \phi_{11})$ form a reduced set of three effective parameters, while the other eigenvectors are functions of one or more of these. In Figure S8, we plot triplets of $\boldsymbol{\phi} = (\phi_1, \phi_5, \phi_{11}) \in \mathbb{R}^3$ colored by the values of Cole's proposed effective parameters, defined in Equation (52). Visual inspection suggests that there is a bijective map, $h : \boldsymbol{\phi} \mapsto \boldsymbol{\beta}$, between the two sets of coordinates.

We used both geometric harmonics (GHs) and neural networks (NNs) to fit $\boldsymbol{\beta} = h(\boldsymbol{\phi})$ and $\boldsymbol{\phi} = h^{-1}(\boldsymbol{\beta})$, achieving a high degree of predictive accuracy in both directions. The mean squared prediction errors are presented in Table S4. For both maps fit via GHs, we computed the Jacobian of the gradient at each input setting. Since values of the analytical effective

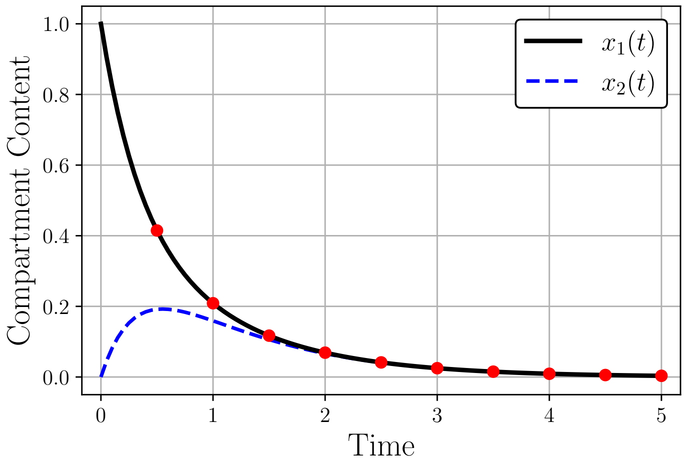

Fig. S7. Dynamic behavior of the model in Equation (50) given reference parameter values $\tilde{\boldsymbol{p}} = (1, 1, 1, 1)$. We observe only $x_1(t)$ at ten equally-spaced times, which are indicated by the red circles.

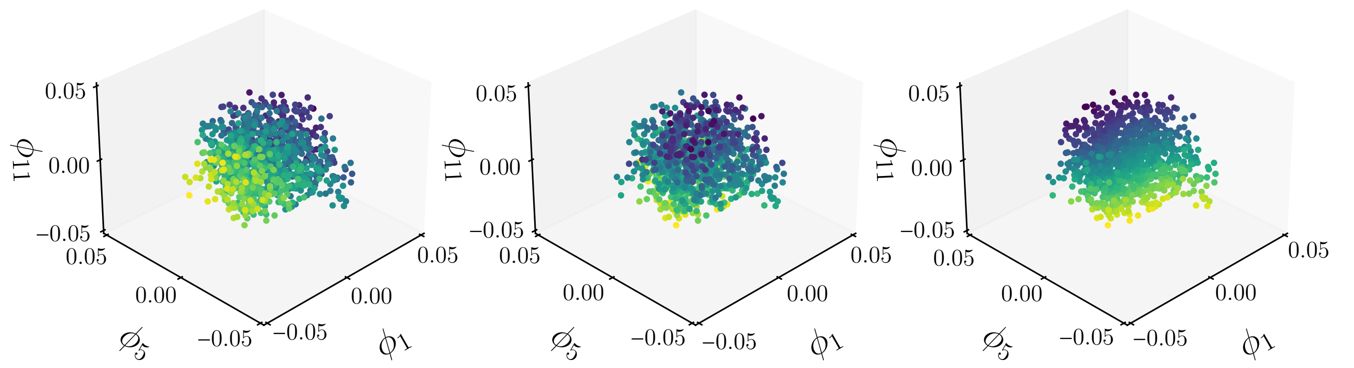

Fig. S8. Our data-driven effective parameters, colored by theoretically proposed $\beta_1, \beta_2, \beta_3$ (left, center, right, respectively). The one-to-one correspondence between data-driven and theoretical parameters is visible.

parameters are approximately two orders of magnitude greater than those of the data-driven parameters, we scale the partial derivatives by the ratio of the corresponding coordinates' standard deviations over the data. This step removes the effects of scale differences between the two spaces. Figure S9 illustrates that the determinants are all of the same sign and are bounded away from zero and infinity.

We also used a conformal autoencoder (CAE) to learn the effective and redundant parameter combinations. In particular, we trained subnetworks for the following three mappings:

$$\text{Encoder:} \quad f_e : \mathbb{R}^4 \to \mathbb{R}^4 : \boldsymbol{p} \mapsto (\boldsymbol{\nu}, \psi)\,, \tag{54}$$

$$\text{Decoder:} \quad f_d : \mathbb{R}^4 \to \mathbb{R}^4 : (\boldsymbol{\nu}, \psi) \mapsto \boldsymbol{p}\,, \tag{55}$$

$$\text{Predictor:} \quad f_p : \mathbb{R}^3 \to \mathbb{R}^{10} : \boldsymbol{\nu} \mapsto \boldsymbol{y}\,. \tag{56}$$

Figure S10 illustrates the trained CAE's ability to reconstruct the original parameter values with a high level of accuracy.

We then sought to parameterize a level set of the redundant parameter ψ. We generate $N'' = 5000$ new parameter vectors, $\{\boldsymbol{p}_i''\}_{i=1}^{N''}$, as before; this time we perturb each component within $\pm 25\%$ of its base value. From each of these points, we use the BFGS algorithm to minimize the objective

$$g(\boldsymbol{p}) = \sum_{i=1}^{10} \Big(y_i(\boldsymbol{p}) - \tilde{y}_i \Big)^2 \,, \tag{57}$$

with stopping criterion $\|\nabla g(\boldsymbol{p})\|_\infty < 10^{-8}$. The resulting minimizers of g are vectors of parameters that achieve the same output as our base point $\tilde{\boldsymbol{p}}$. We compute DMaps on these minimizers and find that they lie on a one-dimensional manifold in the four-dimensional parameter space, corroborating our finding that there are three effective parameters, since $3 + 1 = 4$. We demonstrate in Figure S11 that the DMaps coordinate ψ_1, which parameterizes the level set we discovered by optimizing g, is one-to-one with the redundant conformal coordinate of our CAE. In this case, we evaluated the trained encoder f_e on each of

Table S4. Root-mean-square prediction errors, by geometric harmonics and neural networks, of analytical and data-driven effective parameters for the compartmental model defined in Equation (51).

	$\boldsymbol{\beta} = h(\boldsymbol{\phi})$			$\boldsymbol{\phi} = h^{-1}(\boldsymbol{\beta})$		
	β_1	β_2	β_3	ϕ_1	ϕ_5	ϕ_{11}
GH Train	1.10×10^{-4}	5.26×10^{-4}	4.02×10^{-4}	1.17×10^{-6}	1.61×10^{-5}	7.14×10^{-5}
GH Test	1.87×10^{-4}	9.44×10^{-4}	7.04×10^{-4}	1.62×10^{-6}	2.36×10^{-5}	9.22×10^{-5}
NN Train	1.34×10^{-4}	3.34×10^{-4}	2.98×10^{-4}	2.87×10^{-5}	9.86×10^{-5}	3.96×10^{-4}
NN Test	1.47×10^{-4}	4.85×10^{-4}	3.99×10^{-4}	2.33×10^{-5}	9.85×10^{-5}	3.70×10^{-4}

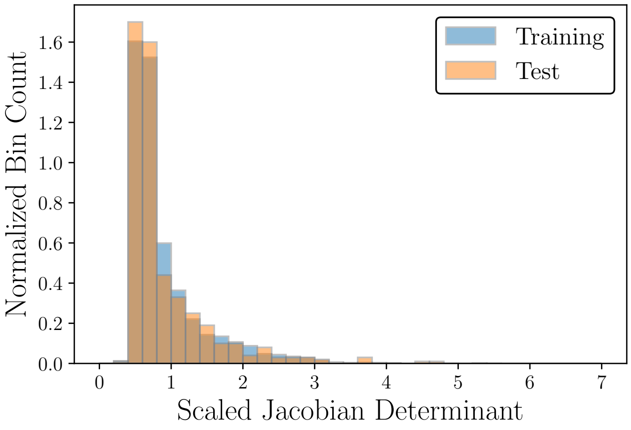

(a)

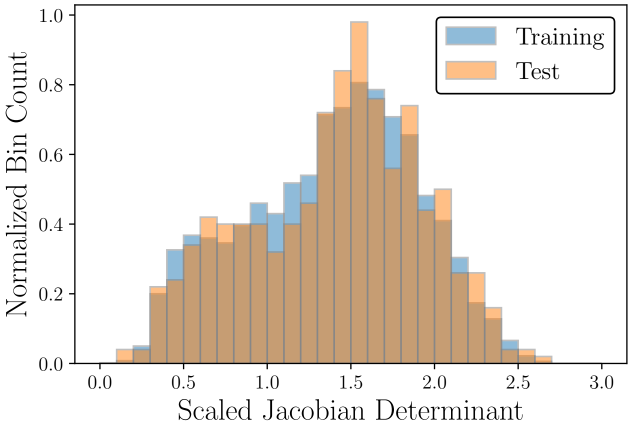

(b)

Fig. S9. Histogram of (a) the quantity $J_h(\boldsymbol{\phi}_i)$ on our data-driven reduced coordinates and (b) the quantity $J_{h^{-1}}(\boldsymbol{\beta}_i)$ on the analytical reduced coordinates, after scaling to account for the order-of-magnitude difference. All bin counts have been normalized such that the plots correspond to empirical probability densities.

the minimizers from our optimization data, which were not used during model training, and compared the fourth conformal coordinate against ψ_1.

6. Transitions between different parametric regimes: A Reaction Engineering Example

Our last example is a static (steady state) problem from chemical kinetics, which allows us to discuss how our data-driven framework identifies the number of effective parameters when the system's response changes its nature (and dimensionality) in different parameter regimes. We consider a first-order catalytic reaction occurring in a spherical pellet with external mass transfer limitations. The parameters are k the reaction rate constant with units of inverse time $[1/T]$, α the characteristic length (pellet radius in the case of a sphere) with units of length $[L]$, D the diffusion coefficient with units of length squared and per time $[L^2/T]$, and k_m the external mass-transfer coefficient with units of length per time $[L/T]$. The output is the pellet production rate, expressed in the form of what is called the *effectiveness factor* η (1, 22) defined as

$$\eta \equiv \frac{R_{jp}}{R_{jb}}; \quad [58]$$

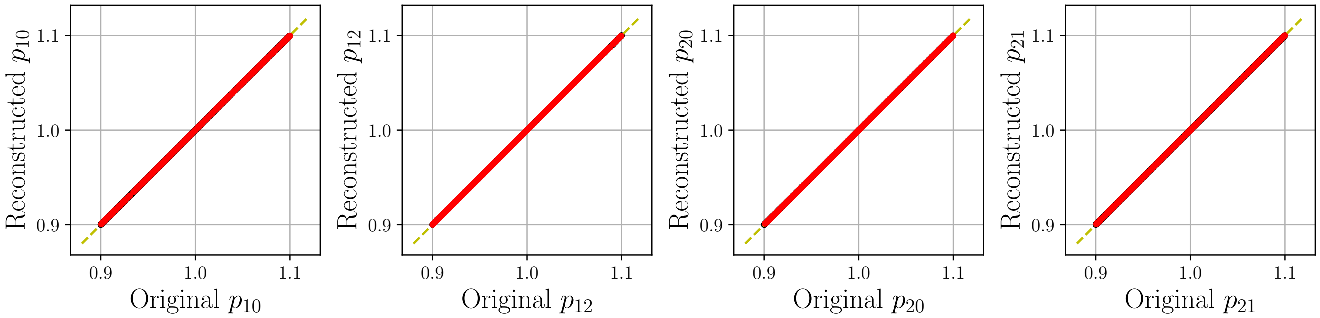

Fig. S10. Comparison of the true parameter values $\boldsymbol{p}$ and the values recovered after applying the trained encoder and decoder, $f_d(f_e(\boldsymbol{p}))$. Test data, in red, overlay training data, in black.

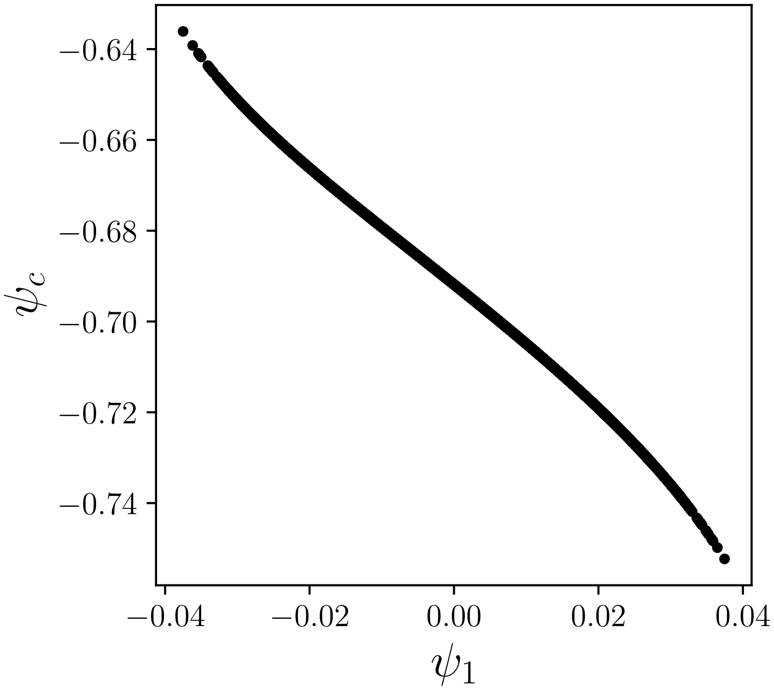

Fig. S11. Comparison of two parameterizations for the redundant parameter of the compartmental model. The DMaps coordinate ψ_1 is obtained from 5000 new parameter vectors, $\boldsymbol{p}_i''$, chosen to achieve the same output behavior, $\tilde{\boldsymbol{y}} = \boldsymbol{y}(\tilde{\boldsymbol{p}})$, as our original base point. Our conformal autoencoder learns the quantity ψ_c as the fourth output of its encoder subnetwork. Values of ψ_c as predicted by the encoder for each $\boldsymbol{p}_i''$ are related to their corresponding DMaps coordinates by a one-to-one mapping.

Here R_{jp} is the pellet's production rate for the j-the species and R_{jb} is the production rate for the the j-th species if the pellet reacted at the bulk concentration of this species both with units of moles per time per unit catalyst volume $[mol/(T \cdot L^3)]$.

It can be shown (1) that

$$\eta = \frac{1}{\Phi}\left[\frac{1/\tanh(3\Phi) - 1/(3\Phi)}{1 + \Phi(1/\tanh(3\Phi) - 1/(3\Phi))/\mathrm{B}}\right], \qquad [59]$$

where Φ is the so-called Thiele modulus, and B is the Biot number. For a first-order reaction, these dimensionless numbers are given by

$$\Phi \equiv \sqrt{\frac{k\alpha^2}{D}}, \qquad \mathrm{B} \equiv \frac{k_m\alpha}{D}, \qquad [60]$$

As can be seen from Equations (59) and (60), the response is effectively one-dimensional and depends on four parameters (k, α, D, k_m). Since dimensional analysis already reduces the original number of parameters of the problem to only two—namely, Φ and B—this allow us to plot and visualize how the response changes for a range of these quantities.

For our computations we generated 10,000 pairs of Φ and B independently and log-uniformly such that $\Phi \in [10^{-2}, 10^6]$ and $\mathrm{B} \in [10^{-4}, 10^8]$ and computed the corresponding reaction rates and overall effectiveness factor values, Equation (59). We

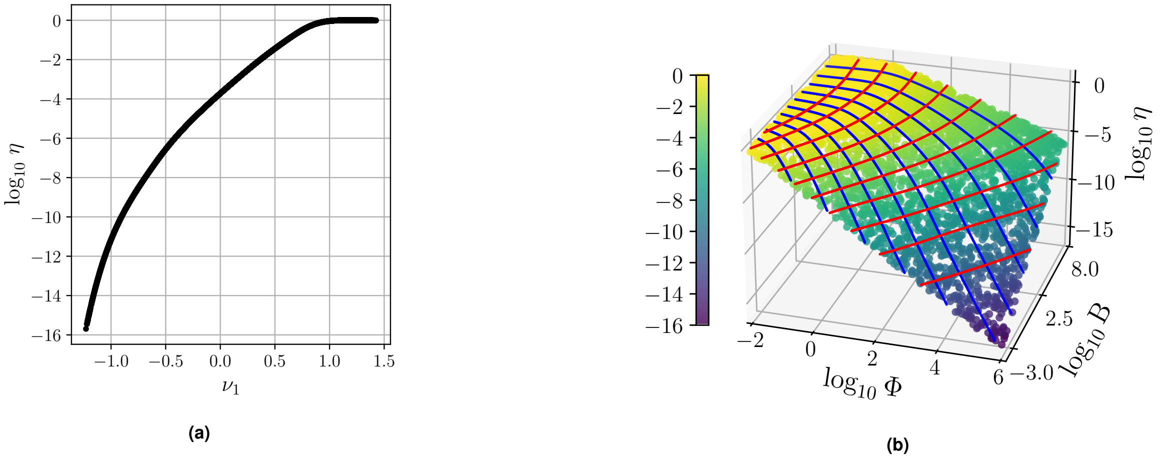

Fig. S12. (a) The effective parameter ν_1, as identified from the network, plotted against the effective factor computed from Equation (59). The true response surface of η is plotted against the parameters Φ and B. The level sets of the meaningful parameter combination are shown with red lines and the redundant parameter combinations with blue lines. The values of η for those level sets were predicted through the behavior estimator network.

trained our Y-shaped conformal autoencoder network by using the same number of hidden layer and activation functions as the network discussed in Section G, but now only two input/bottleneck/output neurons. The effective parameter ν_1 identified by the network, as can be seen in Figure S5 on the left, is one-to-one with the analytical effective parameter η (note also that it "levels out" in the region that the effectiveness factor becomes constant and equal to 1, the so-called reaction-controlled regime). The JSF algorithm applied to this data also leads to a single data-driven effective parameter.

Inspection of Equation (59) clearly shows that there are three qualitatively distinct parameter regimes depending on the magnitude of Φ and B:

$$\eta(\Phi, \mathrm{B}) \approx \begin{cases} \mathrm{B}/\Phi^2 & : \Phi > \max\left(\sqrt{\mathrm{B}}, \mathrm{B}\right) \\ 1/\Phi & : 1 < \Phi < \mathrm{B} \\ 1 & : \Phi < \min\left(\sqrt{\mathrm{B}}, 1\right) \end{cases}. \qquad [61]$$

We find that our framework is capable of capturing regime changes: in the first regime, where $\eta \approx \mathrm{B}/\Phi^2$, the slopes of our level sets shown in Figure S12b show that both parameters affect the output, which therefore depends on a combination of both. In the second regime, where $\eta \approx 1/\Phi$, our effective parameter level sets for η become *parallel to the B number axis*, indicating that the effectiveness factor depends (locally) *only* on Φ. In the third (constant η=1) regime, our effective parameter correctly predicts the constant (the "flattened out" region we already pointed in in Fig. S12).

References

1. JB Rawlings, JG Ekerdt, *Chemical Reactor Analysis and Design Fundamentals.* (Nob Hill Pub, LLC), (2002).
2. E Yeung, et al., Inference of multisite phosphorylation rate constants and their modulation by pathogenic mutations. *Curr. Biol.* **30**, 877–882 (2020).
3. JB Tenenbaum, V de Silva, JC Langford, A global geometric framework for nonlinear dimensionality reduction. *Science* **290**, 2319–2323 (2000).
4. ST Roweis, LK Saul, Nonlinear dimensionality reduction by locally linear embedding. *Science* **290**, 2323–2326 (2000).

5. M Belkin, P Niyogi, Laplacian eigenmaps for dimensionality reduction and data representation. *Neural Comput.* **15**, 1373–1396 (2003).
6. RR Coifman, S Lafon, Diffusion maps. *Appl. Comput. Harmon. Analysis* **21**, 5–30 (2006).
7. SS Lafon, Ph.D. thesis (Yale University, New Haven, CT) (2004).
8. CJ Dsilva, R Talmon, RR Coifman, IG Kevrekidis, Parsimonious representation of nonlinear dynamical systems through manifold learning: A chemotaxis case study. *Appl. Comput. Harmon. Analysis* **44**, 759–773 (2018).
9. A Holiday, et al., Manifold learning for parameter reduction. *J. Comput. Phys.* **392**, 419–431 (2019).
10. C Fowlkes, S Belongie, J Malik, Efficient spatiotemporal grouping using the Nyström method in *Proceedings of the 2001 IEEE Computer Society Conference on Computer Vision and Pattern Recognition.* (IEEE), Vol. 1, pp. I–I (2001).
11. RR Coifman, S Lafon, Geometric harmonics: A novel tool for multiscale out-of-sample extension of empirical functions. *Appl. Comput. Harmon. Analysis* **21**, 31–52 (2006).
12. EJ Nyström, *Über die Praktische Auflösung von Linearen Integralgleichungen mit Anwendungen auf Randwertaufgaben der Potentialtheorie.* (Akademische Buchhandlung), (1929).
13. H Wendland, *Scattered Data Approximation.* (Cambridge university press) Vol. 17, (2004).
14. MK Transtrum, P Qiu, Model reduction by manifold boundaries. *Phys. Rev. Lett.* **113**, 098701 (2014).
15. JE Marsden, MJ Hoffman, *Elementary Classical Analysis.* (Macmillan), (1993).
16. AF Brouwer, MC Eisenberg, The underlying connections between identifiability, active subspaces, and parameter space dimension reduction. *arXiv preprint arXiv:1802.05641* (2018).
17. RA Horn, CR Johnson, *Matrix Analysis.* (Cambridge University Press), (2012).
18. F Dietrich, O Yair, R Mulayoff, R Talmon, IG Kevrekidis, Spectral discovery of jointly smooth features for multimodal data. *SIAM J. on Math. Data Sci.* **4**, 410–430 (2022).
19. DJ Cole, *Parameter Redundancy and Identifiability.* (CRC Press, Boca Raton, FL), (2020).
20. M España, ID Landau, Reduced order bilinear models for distillation columns. *Automatica* **14**, 345–355 (1978).
21. JA Jacquez, Compartmental modeling. *IFAC Proc. Vol.* **21**, 31–37 (1988) IFAC Symposium on Modelling and Control in Biomedical Systems, Venice, Italy, 6-8 April.
22. EW Thiele, Relation between catalytic activity and size of particle. *Ind. & Eng. Chem.* **31**, 916–920 (1939).
